# State-sponsored agenda setting: Measuring the impact of narrative laundering

**DOI:** 10.1073/pnas.2534920123

**Published:** 2026-09-04

**Authors:** Patrick L. Warren, Darren L. Linvill, Camille J. Saucier

**Affiliations:** ^a^ Media Forensics Hub, Watt Family Innovation Center, Clemson University, Clemson, SC 29634; ^b^ John E. Walker Department of Economics, Clemson University, Clemson, SC 29634; ^c^ Department of Communication, Clemson University, Clemson, SC 29634

**Keywords:** agenda setting, narrative laundering, foreign information manipulation and interference, disinformation, social media

## Abstract

Foreign information manipulation and interference (FIMI) is an important part of the digital information ecosystem, yet its effects are poorly understood. Most research analyzing FIMI to date is descriptive, exposing efforts to erode trust, promote favorable narratives, and target specific communities. Existing work evaluating the impacts of FIMI contrasts those directly exposed to FIMI messaging (by viewing the original messages) with those who were not, finding little evidence of changes in beliefs or behavior. Rather than persuading individuals directly, we argue FIMI often operates through agenda-setting processes that shape which issues and narratives dominate public discourse. A key component of this process is narrative laundering, whereby misleading content is scrubbed of its origins and circulated into mainstream channels. This study examines the Russian-aligned Storm-1516 campaign, which uses state media, fabricated sites, and paid influencers to elevate false narratives, especially those that implicate Ukrainian President Volodymyr Zelenskyy in corrupt acts. By analyzing the dissemination of these narratives, we assess how Storm-1516 shifts the public agenda, illustrating how foreign influence can exert substantial effects on information environments even when individual-level persuasive effects may be limited.

## Significance

Research examining the impacts of foreign malign influence has focused on its effects on voter behavior and political attitudes. No change in these outcomes has been found. There is evidence, however, that changes to voter behavior and beliefs may not be the only goal of foreign information manipulation and interference. One goal of past Russian influence operations was to serve an agenda-setting function by directing the public’s attention to particular issues and shaping how they were discussed. By focusing on agenda-setting effects rather than direct attitudinal effects we can understand how influence operations achieve intended goals without driving broad changes to individuals’ political attitudes. We examine a Russian narrative-laundering campaign to measure and demonstrate the impact of such influence campaigns.

Foreign information manipulation and interference (FIMI) has become part of the fabric of our digital world. Work aimed at better understanding FIMI has examined campaigns originating from a range of state actors, predominantly Russia (1), China (2), and Iran (3). The plurality of this research is descriptive—aimed at better understanding the tactics and strategies employed by state actors. Such work has shown how foreign campaigns have endeavored to undermine trust in science (4), promote narratives favorable to a nation-state (5), and tailor messages for specific communities (6). Few studies, however, have attempted to measure the impact of these campaigns and, thus, better understand their implications. This is, in part, due to methodological obstacles and data limitations that make such research difficult. As a result, we still do not fully know if and how digital FIMI campaigns matter, despite significant efforts to describe them.

There is a clear danger in overstating the impact of FIMI given the potential to embolden bad actors and reinforce their messaging. As Belogolova et al. note, fixation on foreign disinformation’s potential effects may foster a “conspiratorial outlook, in which shadowy enemies are supposedly creating wedge issues, dissenters are merely parroting foreign spies, and trust in open democratic debate is eroded” (7). That said, given that we do not have a full understanding of how and the degree to which FIMI affects organic online discourse, it is difficult to say what is an overstatement and what may be a clear-eyed assessment.

Research examining the impacts of FIMI to date has focused on its effects on voter behavior and political attitudes (8, 9). No change in either type of outcome has been found. There is evidence, however, that changes to voter behavior and beliefs may not be the only, or even primary, goal of some FIMI. Research suggests that one goal of past Russian influence operations was to serve an agenda-setting function (10).

Agenda setting offers a compelling framework for understanding how state-affiliated influence operations impact public discourse. Agenda-setting theory posits that media coverage shifts which issues attract attention and are salient to target audiences (11), shaping which topics audiences consider important and how these issues are discussed (12). By selectively highlighting certain characteristics and implications of a topic while downplaying others, media can influence how audiences cognitively process content (13). A distinct but related concept, agenda-building, refers to the process by which various groups, such as politicians, companies, and foreign governments, influence which issues are selected for media coverage (14, 15). This perspective facilitates an evaluation of the effectiveness of foreign influence operations beyond measuring microlevel attitudinal or behavioral change, instead moving to the macrolevel information environment (16).

In this study, we examine a Russian-aligned FIMI campaign to evaluate its success in propagating several specific narratives in public discourse and, in turn, shifting the salience of those narratives within the public agenda. By focusing on agenda-setting effects rather than direct attitudinal effects, we can better understand how state-affiliated influence operations achieve intended goals without necessarily driving broad changes to individuals’ political attitudes. For instance, while the success of a campaign could be measured by assessing how many people believe a state-sponsored narrative or how they act on those beliefs, such assessments could instead focus on how effectively these campaigns shift which issues occupy public attention because, for instance, that public attention may influence the policy agenda.

This study uses agenda setting as a lens and shows that FIMI can serve to substantially alter the way in which users discuss and engage with an issue online. Further, we demonstrate that FIMI’s impact on an issue can be maintained over time through the use of repeated interventions.

This move to understanding FIMI through an agenda-setting lens is important for two nonmutually exclusive reasons. First, it offers an argument for why estimates of the persuasive or behavioral impacts of direct exposure to original FIMI messaging might be small. Given the indirect nature of many agenda-setting effects, it may be difficult to distinguish users who have been exposed to FIMI from those who have not within a complex media ecosystem. Such a comparison is difficult because so much exposure to narratives is potentially indirect, where you do not see the original message but instead see the narrative repeated by a third party in their own words. As a result, work that compares the beliefs and behaviors of those directly subjected to FIMI messaging to those who were not can only estimate the impact of direct treatment. But the impact of direct exposure may be only a small part of the overall impact on the beliefs and behaviors of those subjected to the change in the information environment following the FIMI intervention. Conclusions that FIMI is ineffective that draw exclusively from evidence concerning these *direct* effects (8, 9) could substantially understate its impact.

A second insight the agenda-setting lens offers is to note that behavioral and attitudinal change may not even be necessary for FIMI to achieve its objectives. Previous research has shown that various agendas, including the media agenda and the agenda of policymakers, are closely linked and can influence one another (17). Research on Twitter found that legislators are more likely to follow citizens’ public agenda than to lead and set their own and are particularly responsive to the views expressed by supporters (18). In short, it may not be necessary for Russian FIMI to cause genuine changes in belief if they can simply cause policy makers to perceive their supporters as holding a specific view, because that view is discussed prominently. Agenda setting suggests, in other words, that simply changing public discourse can lead to policy change without meaningful change in individual beliefs.

One specific way that FIMI can strategically exploit agenda-setting mechanisms is through narrative laundering, the process of circulating information from unverified sources into the mainstream discourse (19). Through narrative laundering, the origins of false or misleading information are concealed while simultaneously elevating this content, typically in a manner that appears organic (19, 20). Narrative laundering has been used by Russian actors since the Soviet era (21). Scholars describe the process as proceeding through three stages, adapted from our understanding of money laundering: placement, layering, and integration. Placement involves the initial introduction of narratives into the information ecosystem, layering moves the narrative purposefully from less credible origins to increasingly legitimate-seeming sources, and integration occurs when a narrative is finally shared by genuine audiences who circulate it organically. FIMI leverages narrative-laundering processes to inject false narratives, including those identified in the study sample, into public discourse.

Storm-1516 is an ongoing narrative-laundering campaign attributed to Russian-aligned actors that was first brought to broad public attention by the BBC and Clemson University’s Media Forensics Hub (22) and subsequently dubbed “Storm-1516” by Microsoft’s Threat Analysis Center (23). This campaign uses techniques practiced by the Russian KGB during the Cold War, enhanced by modern digital technologies. Storm-1516 leverages a broad mix of distribution methods. These include compliant or compensated foreign media, Russian state media, websites fabricated to appear as genuine Western media outlets (22), and, perhaps most importantly, paid social media influencers (24).

Russia’s Internet Research Agency was known to have relied on inauthentic social media accounts to spread desired narratives. In contrast, Storm-1516 has leveraged paid influencers. A detailed analysis conducted by Viginum, a unit within the French Secretariat-General for National Defense and Security, examined the spread of 77 distinct Storm-1516 narratives (25). The report found that although the campaign occasionally used individual inauthentic accounts for initial content placement, subsequent amplification primarily relied on layering conducted by paid influencer accounts with substantial online followings. Viginum did not identify widespread use of coordinated inauthentic social media accounts or bot networks to promote Storm-1516 narratives. Instead, social media discourse around these narratives was driven through engagement with influencers remunerated by the campaign, reflecting tactics commonly used in mainstream digital marketing (26).

Much of Storm-1516’s activities relate to Russia’s broader FIMI efforts to undermine international support for Ukraine’s military defense. Here, we examine narratives placed between July 2023 and December 2024. At least 65 distinct narratives have been attributed to Storm-1516 in this period (25). thirty-seven of these narratives target Ukraine, of which 21 specifically target Ukrainian President Volodymyr Zelenskyy or his wife Olena Zelenska.

One metanarrative promoted by Storm-1516 sought to portray the Zelenskys as both morally and financially corrupt. During the study period, 11 false narratives were identified, in which the FIMI campaign described a variety of extravagant, fictional purchases the couple made, implying that the Zelenskys used Western aid funds to fuel their own profligate lifestyle—ranging from purchasing multimillion-dollar yachts to acquiring a vineyard in France. Our study uses the introduction of these false narratives to better understand Storm-1516’s impact on agenda-setting concerning the Russian invasion of Ukraine and on individual search behavior. Specifically, we ask:

1. RQ1: How, if at all, has Storm-1516 narrative laundering impacted social media discourse around Ukrainian President Volodymyr Zelenskyy and his wife Olena Zelenska.
2. RQ2: How, if at all, has Storm-1516 narrative laundering impacted internet search behavior around Ukrainian President Volodymyr Zelenskyy and his wife Olena Zelenska.

To trace the impact of the agenda-setting element of the Storm-1516 campaign, we conducted a series of event-study analyses to identify the effect of the introduction of laundered narratives on the mix of posts on the social media platform X about Zelenskyy. Similarly, we examine the effect of the introduction of these false narratives on the mix of internet search behavior about Zelenskyy.

We find that the impact of these interventions is substantial. There was practically no discussion of keywords related to the false narratives in the weeks before they were introduced. In the week immediately after a false narrative was first introduced, the average share of discussion about Zelenskyy on X that contained narrative-relevant keywords rose to 7.5% and remained at 1% of the discourse in the subsequent week. Using an LLM to code the share of the Zelenskyy-related posts on X that relate to a general Zelenskyy-corruption metanarrative, we see the discussion associating Zelenskyy with corruption jump from 10% of online discourse in the weeks before the false narrative was introduced to about 17% during the week it was disseminated. Finally, the false narratives rose from nothing to over 5% of Zelenskyy-related searches in the week following their introduction, before falling to 2.5% in the second week and to just below 1% in the third week.

## Empirical Methods

Between July 1, 2023, and December 31, 2024, Storm-1516 introduced 11 false narratives into the public discourse alleging that Volodymyr Zelenskyy or his wife, Olena Zelenska, had acquired expensive luxury items or real estate (see *SI Appendix*, Fig. S1 for details of each narrative). Our goal is to measure the impact of these interventions on multiple dimensions of the public discourse surrounding Zelenskyy in the weeks following their introduction. Specifically, we perform event-study analyses to measure the relationship between the introduction of these narratives and changes in three outcomes: 1) a keyword-based proxy for the share of the discourse about Zelenskyy/a on X related to these specific false narratives, 2) a LLM-based proxy for the share of discourse about Zelenskyy/a on X involving accusations or implications of corruption, more generally, and 3) a keyword-based proxy for the share of Google Search queries about Zelenskyy/a related to these narratives (data and replication scripts available at ref. 27).

### Narrative Discussion Shares.

For each of the 11 narratives, we defined a Boolean keyword query that captures a large share of posts that shared, debated, or interacted with this narrative. The full queries are reported in *SI Appendix*, Fig. S1. Using Sprinklr, a social-media listening platform, we collected daily counts of posts on the social-media platform X that satisfied each query, along with a broader query capturing all mentions of Zelenskyy in either the masculine or feminine form (“Zelensky/Zelenskyy/Zelenska”), between July 1, 2023, and December 31, 2024. Beginning with the date in which each narrative was introduced, we calculated the narrative discussion share as the total number of posts mentioning both the narrative and Zelenskyy/Zelenska, divided by the total number of posts mentioning Zelenskyy/Zelenska during that week. We calculated *this measure* for the 4 wk before, the week of, and the week after the narrative was introduced. Our event study analysis was run on these 99 observations (9 wk for each of the 11 narratives).

### Narrative Search Shares.

For each of the 11 narratives, we used variants of the queries employed to assess discourse on X (*SI Appendix*, Fig. S1), to track worldwide Google Trends Search scores for each narrative as well as a broader query that included all mentions of Zelenskyy. We collected data for the week in which each narrative was introduced, the 4 wk before, and the 4 wk after its introduction. For each narrative, we calculated the narrative search share for each included week as the ratio of the weekly worldwide search-interest score for the narrative to the corresponding search-interest score for Zelenskyy. Our event study analysis was again run on these 99 observations (9 wk for each of the 11 narratives).

Our keyword-based proxies will necessarily undercount the true number of posts or searches addressing the specific narratives. It is impossible to capture all the possible words users might use to discuss these narratives across the various languages in which they might be discussed. We believe our proxies captured the great majority of each narrative, however, as these narratives originated in English and often used highly specific wording (e.g., “Bugatti” or “Cartier”). Nonetheless, findings should be interpreted as providing a lower-bound estimate of the proportion of discourse surrounding each narrative.

### Corruption Metanarrative Shares.

To capture the share of social-media discourse accusing or implying that Zelenskyy is corrupt, we collected a random sample of 40,000 posts mentioning Zelenskyy in either the masculine or feminine form between July 1, 2023, and December 31, 2024. We then used a large language model (GPT-4o mini) as a text classification tool. Each sampled post was provided to the model as text only, without attached images, linked content, or conversational thread context. The model was instructed via a fixed few-shot prompt to assign one of three labels: 1) supports or implies the Zelenskyy corruption metanarrative, 2) fact-checks or debunks that narrative, or 3) unrelated. The full prompt is reproduced in *SI Appendix*, Fig. S3.

To assess the accuracy of this procedure, two human research assistants manually coded a random validation subset of 299 posts. Disagreements were reconciled, and the adjudicated human labels were compared with the model’s labels. Agreement was substantial (three-class accuracy = 0.729, kappa = 0.552), with particularly strong performance in distinguishing posts that implicated corruption from those that did not (corruption-label precision = 0.867; recall = 0.580). Most disagreements involved posts that required contextual information unavailable to the model, but available to the human coders, such as images, prior posts within a thread, or contemporaneous news context.

Using these postspecific LLM classifications, we estimated the share of posts containing corruption allegations for the week beginning on the day each narrative was introduced, as well as the 4 wk prior and the 4 wk after introduction. Our event study analysis was again run on these 99 observations (9 wk for each of the 11 narratives).

Relative to the keyword-based approach, this LLM-classified metric is a much broader measure indicating whether a post contains an accusation or implication of corruption. It allows for substantial flexibility in word choice, language, and specificity, while also allowing us to exclude fact-checks of claims of corruption. In addition, the measure captures all corruption-related accusations, rather than the specific false narratives examined. Thus, if the injection of false narratives simply displaces existing or unrelated claims of corruption, we can estimate the net impact of the intervention on the overall corruption *metanarrative*.

### Event Studies.

Our general approach for estimating the impact of these interventions is a series of event-study analyses (28). For each outcome variable, represented by $y_{it}$, the relative share for narrative *i* = 1, 2, …, 11 in week *t* = −4, −3, −2, −1, 0, 1, 2, 3, 4, we run a regression of the form:

$$\begin{array}{c} y_{it}\;=\;a_{i}\;+\;B_{0}\;Week0_{it}\;+\;B_{1}\;Week1_{it}\;+\;B_{2}\;Week2_{it}\;\\ \;+\;B_{3}\;Week3_{it}\;+\;B_{4}\;Week4_{it}\;+\;e_{it}, \end{array}$$

where $a_{i}$ is a narrative-level fixed effect and the *$B_{j}$* coefficients correspond to indicator variables for Weeks 0 to 4, where Week 0 represents the week in which the narrative was first published by Storm-1516. We estimate these coefficients using OLS regression, accounting for heteroscedasticity when estimating SE (29). We also report results for two limited-dependent-variable variants of this model, beta and continuous-logit regressions, in *SI Appendix*, Table S4. Results are substantively and statistically similar to the OLS estimates. *SI Appendix*, Table S6 presents an alternative permutation-based approach to the estimation of SE, which may be more robust to our small sample. The main Week 0 results remain strongly significant under this alternative specification.

The fixed effects ($a_{i}$) represent the baseline proxy rate for narrative *i* in the 4 wk before the intervention. Our primary interest lies in the coefficients $B_{j}$, which represent how much the proxy rate increases or decreases in week *j* of the intervention, relative to the 4 wk prior. To the extent that no other relevant interventions affecting the dependent variable co-occur with the Storm-1516 false-narrative injections, this approach provides evidence of the causal effect of the intervention on the share of Zelenskyy-relevant discourse containing a narrative proxy. We present results for varying preintervention windows, in *SI Appendix*, Table S3. Results are substantively and statistically similar to those obtained using the 4-wk window.

## Results

**Fig. 1.**
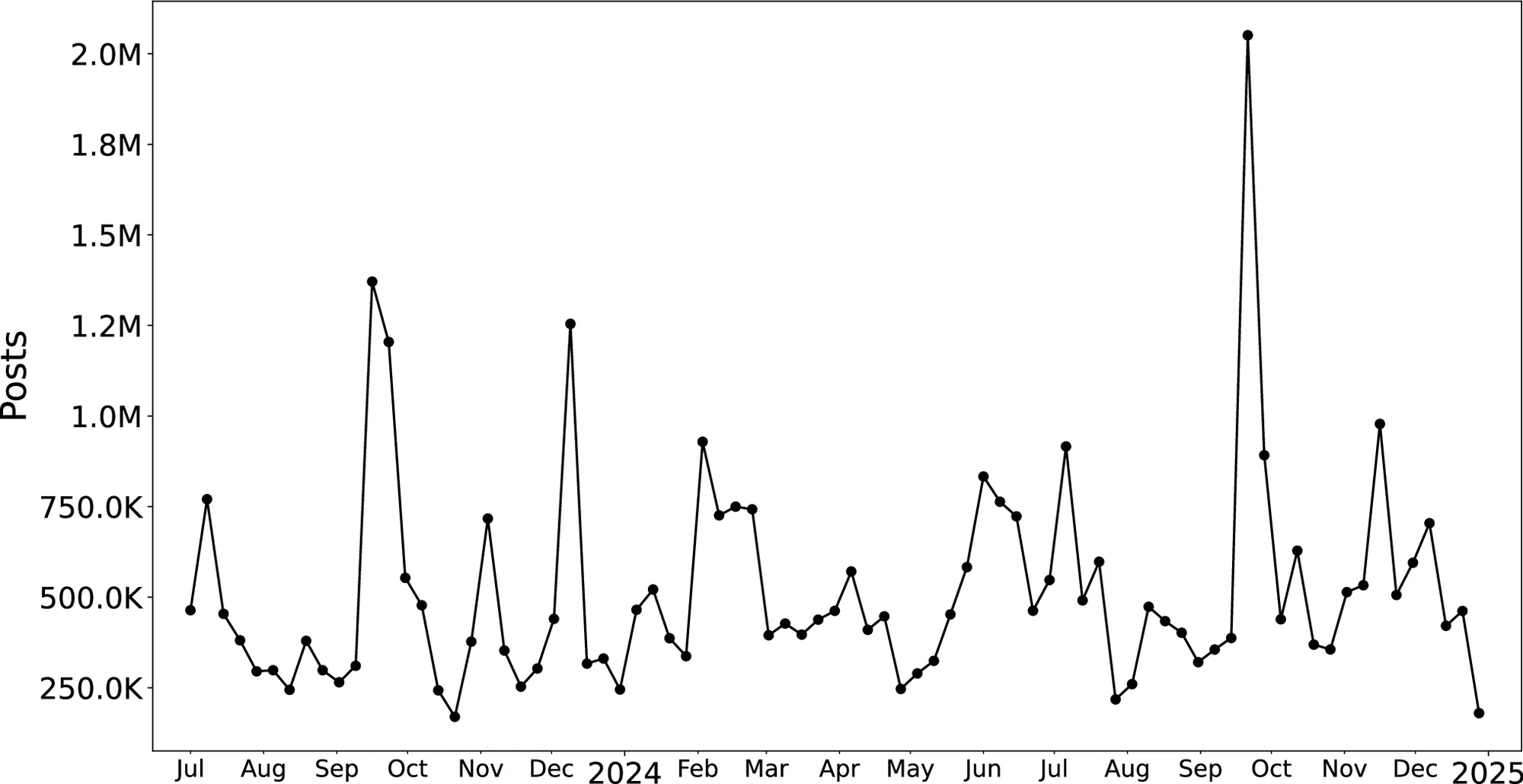
Weekly count of posts on X that include the words “Zelenskyy,” “Zelensky,” or “Zelenska,” July 1, 2023 to Dec 31, 2024.

Over the 18-mo data collection period, the number of posts on X that included the words “Zelensky,” “Zelenskyy,” or “Zelenska” averaged approximately 75,000 per day, although these counts varied dramatically with the news cycle around the war in Ukraine. The highest-volume days occurred on September 26 and 27, 2024, during President Zelenskyy’s visit to Washington, D.C., when mentions reached approximately 400,000 and 570,000 posts, respectively. The lowest-volume days were October 21, 2023, and January 5, 2024, with approximately 15,000 and 16,000 posts, respectively. Fig. 1 displays the full time series of the Zelenskyy-relevant posts, aggregated by week (Sunday to Saturday).

**Fig. 2.**
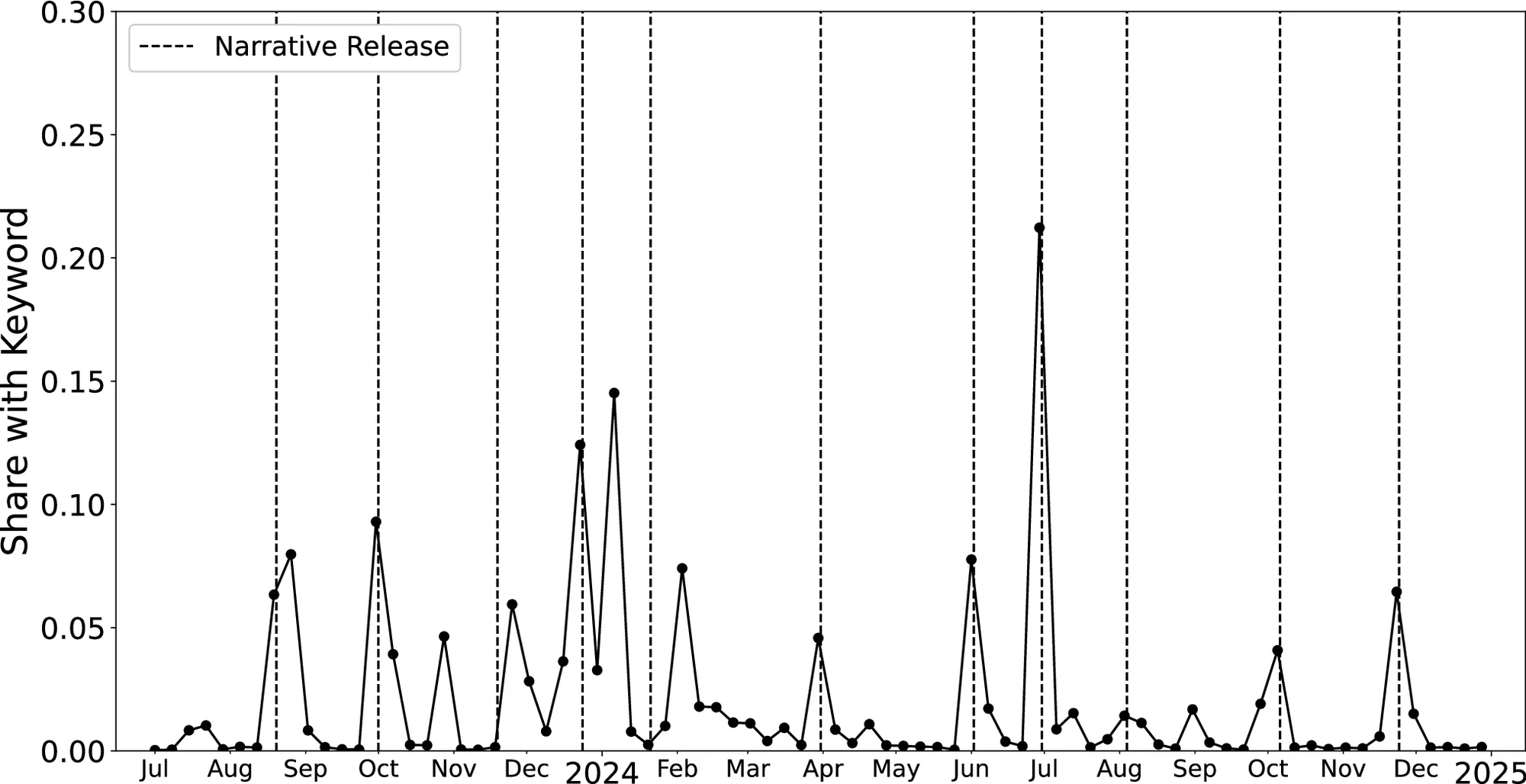
Weekly share of posts on X that include the words “Zelenskyy,” “Zelensky,” or “Zelenska” that also contain one of the Storm-1516 narrative keywords, and timing of Storm-1516 narrative releases, July 1, 2023 to Dec 31, 2024.

With regard to RQ1, we find that the Storm-1516 campaign successfully introduced new narratives accusing Zelenskyy of corruption into discourse on X. The solid line in Fig. 2^*^ depicts a full-time series of the weekly share of Zelenskyy-related posts containing *any* of the narrative-qualifying keywords. Vertical dotted lines indicate the weeks in which each narrative was first released (see *SI Appendix*, Fig. S2 for separate timelines for each false narrative). For all 11 narratives, the share of posts related to the narratives increased in the weeks immediately following their release. In several instances, the share of posts containing the qualifying keywords jumped from zero to 5%, 10%, or even 20% of the total Zelenskyy-related discourse (see *SI Appendix*, Fig. S6 for narrative-by-narrative estimates).

To further investigate RQ1, we measured the share of the overall discussion on X containing keywords that reference each Zelensky narrative and tracked those shares during the 4 week preceding the narrative’s introduction, the week of introduction, and the four subsequent weeks. As an example, for all posts on X that include the words “Zelensky,” “Zelenskyy,” or “Zelenska,” we examined the share of posts that also include the word “Bugatti” before the introduction of this narrative, during the week it was introduced (July 1, 2024), and how that share changed in the weeks that followed.

The results of this analysis are presented in the first column of Table 1 (“X Narrative Share”). Across our 11 narratives, discussion containing narrative-related keywords was substantively and statistically insignificant on average prior to the narratives’ introduction, accounting for less than 0.1% of the discourse around Zelenskyy, in the 4-wk preintervention period. In the week beginning the day the narratives were introduced, the share of the discussion about Zelensky that included those keywords rose by 7.6 percentage points (*p* < 0.01). The elevated share of the false narrative in discussion persists, in a small but statistically significant way, into the first week following the introduction of the narrative. During the first week postintroduction, we observed a 1 percentage-point increase (*p* < 0.01) over the baseline level. The rate remained elevated in the second week postintroduction, although the variance in shares was high enough that we cannot reject a null relationship. By the third and fourth weeks postintroduction, discussion of the narrative fell back down to levels that were indistinguishable from the base rate. These results are robust across the pretreatment windows ranging from 2 to 6 wk (see estimates in *SI Appendix*, Table S3), and the week-0 effects were robust to permutation-based statistical inference (see estimates in *SI Appendix*, Table S6).

**Table 1.** Event-study analysis of the relationship between narrative introduction and proxies of narrative impact

Time period	X narrative share	X corruption share	Google trend share
Base rate	0.000	0.102^**^	0.001
	(0.001)	(0.008)	(0.002)
0 wk after	0.076^**^^,^^+^	0.068^**^^,^^+^	0.053^*^^,^^+^
	(0.017)	(0.010)	(0.024)
1 wk after	0.009^**^	−0.006	0.025^*^
	(0.003)	(0.014)	(0.012)
2 wk after	0.016	−0.006	0.008^*^
	(0.009)	(0.013)	(0.003)
3 wk after	0.003	0.020	0.004
	(0.004)	(0.017)	(0.002)
4 wk after	0.003	0.007	0.003
	(0.004)	(0.019)	(0.003)

Note: *n* = 99 in all regressions. SE are robust to heteroskedasticity.

^*^p < 0.05.

^**^p < 0.01.

^+^Permutation null-hypothesis rejection *P* < 0.01.

To investigate whether the rise in observed keyword shares represented a true increase in discourse accusing Zelenskyy of corruption, we used an LLM (GPT 4o) to code a random sample of 40,000 posts from the sample period that included any variant of the name Zelenskyy/a. The LLM indicated whether each post 1) referenced or accused Zelenskyy or his family of financial corruption (the corruption metanarrative), 2) fact-checked or debunked such accusations, or 3) was unrelated to the metanarrative. Over the full study period, 10.3% of messages were coded as supporting the Zelenskyy corruption metanarrative, while 0.3% were coded as fact-checking the metanarrative. The remaining posts were unrelated.

These shares varied over time. If discussion of the false narratives merely supplanted other references to corruption, or if the conversation was dominated by fact-checking of those narratives, we would expect no change in the share of posts related to the corruption metanarrative following the introduction of the false narratives. Conversely, if the introduction of the false narratives raised the prominence of the Zelenskyy corruption metanarrative, we would expect to see that share increase in the weeks after they were introduced.

**Fig. 3.**
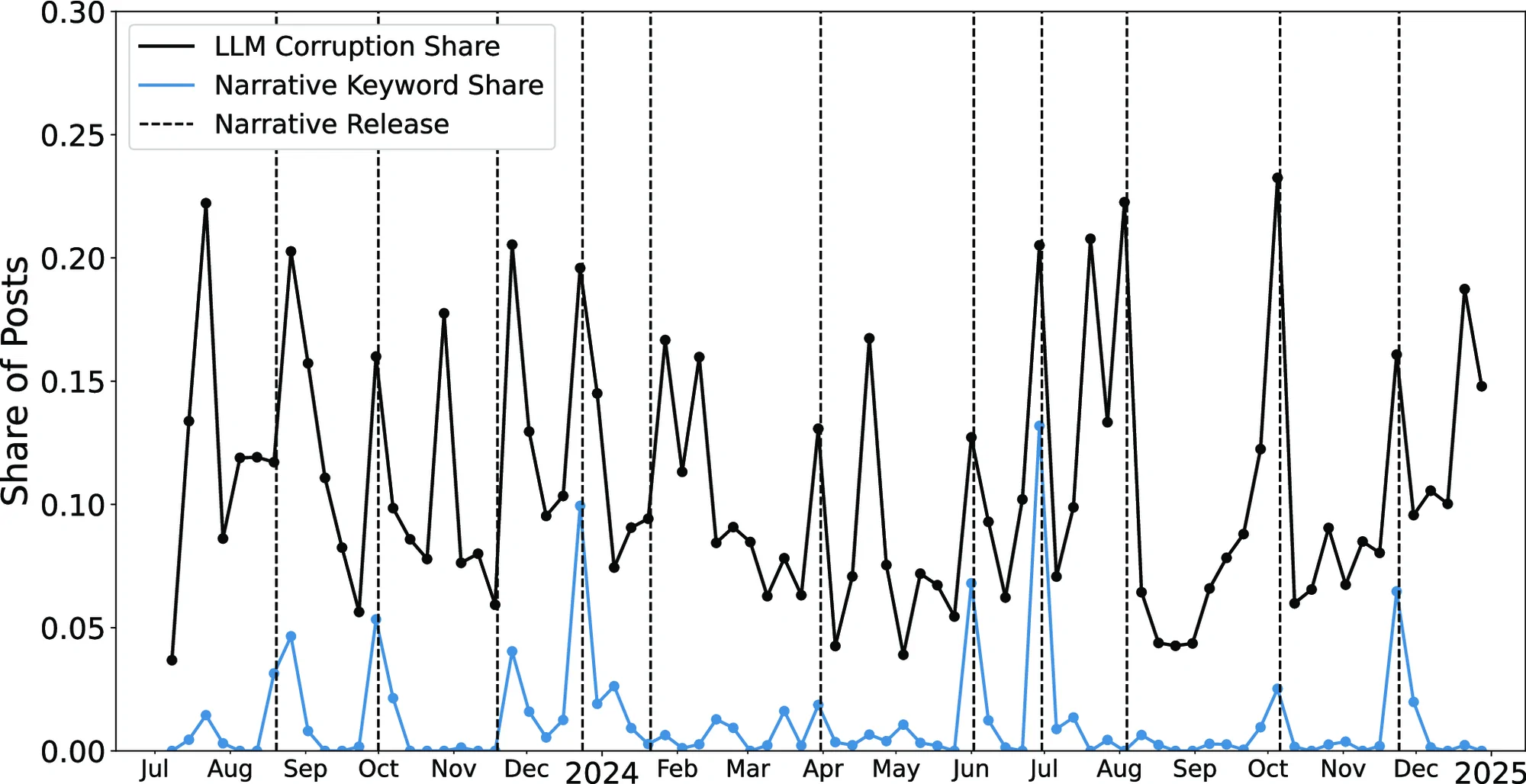
Weekly share of posts on X that include the words “Zelenskyy,” “Zelensky,” or “Zelenska” that also contain one of the Storm-1516 narrative keywords, share that were coded as implying corruption, and the timing of Storm-1516 narrative releases, July 1, 2023 to Dec 31, 2024.

Results suggest that dissemination of the false narratives led to greater overall discussion of corruption, rather than simply altering how corruption was discussed. The upper black line in Fig. 2 presents a time series of the weekly share of messages that were coded as relating to the Zelenskyy corruption metanarrative, while the lower blue line shows the share of sampled posts that contain one of the narrative keywords. Vertical dotted lines denote the dates the Storm-1516 narratives were released. The 11 narrative releases that preceded spikes in the share of messages with narrative-relevant keywords also precede spikes in the share of messages implicating Zelenskyy in corruption (Fig. 3^†^). In fact, in several instances, such as the January and April 2024 narratives, increases in the corruption metanarrative substantially outpaced the narrow keyword metric.

These results do not imply that the Storm-1516 narratives were the sole catalysts of messages implicating Zelenskyy in corruption. The base rate of weekly posts containing corruption-related content is greater than the base rate of posts containing narrative-related keywords, and several spikes in corruption-related discourse occur in the absence of Storm-1516 narratives. Nevertheless, the relationship between the introduction of Storm-1516 narratives and the share of posts involving corruption allegations is apparent.

The results of this analysis are presented in the second column of Table 1 (“X Corruption Share”). On average, there was substantial discussion of corruption during the 4 wk before the false narratives were introduced, with approximately 10% of posts about Zelenskyy coded as relating to corruption. In the week beginning the day the false narratives were introduced, that share rose 6.8 percentage points (*p* < 0.01). This elevated rate did not persist into future weeks, however, as the share of corruption-related posts returned to levels that were indistinguishable from the baseline.

**Fig. 4.**
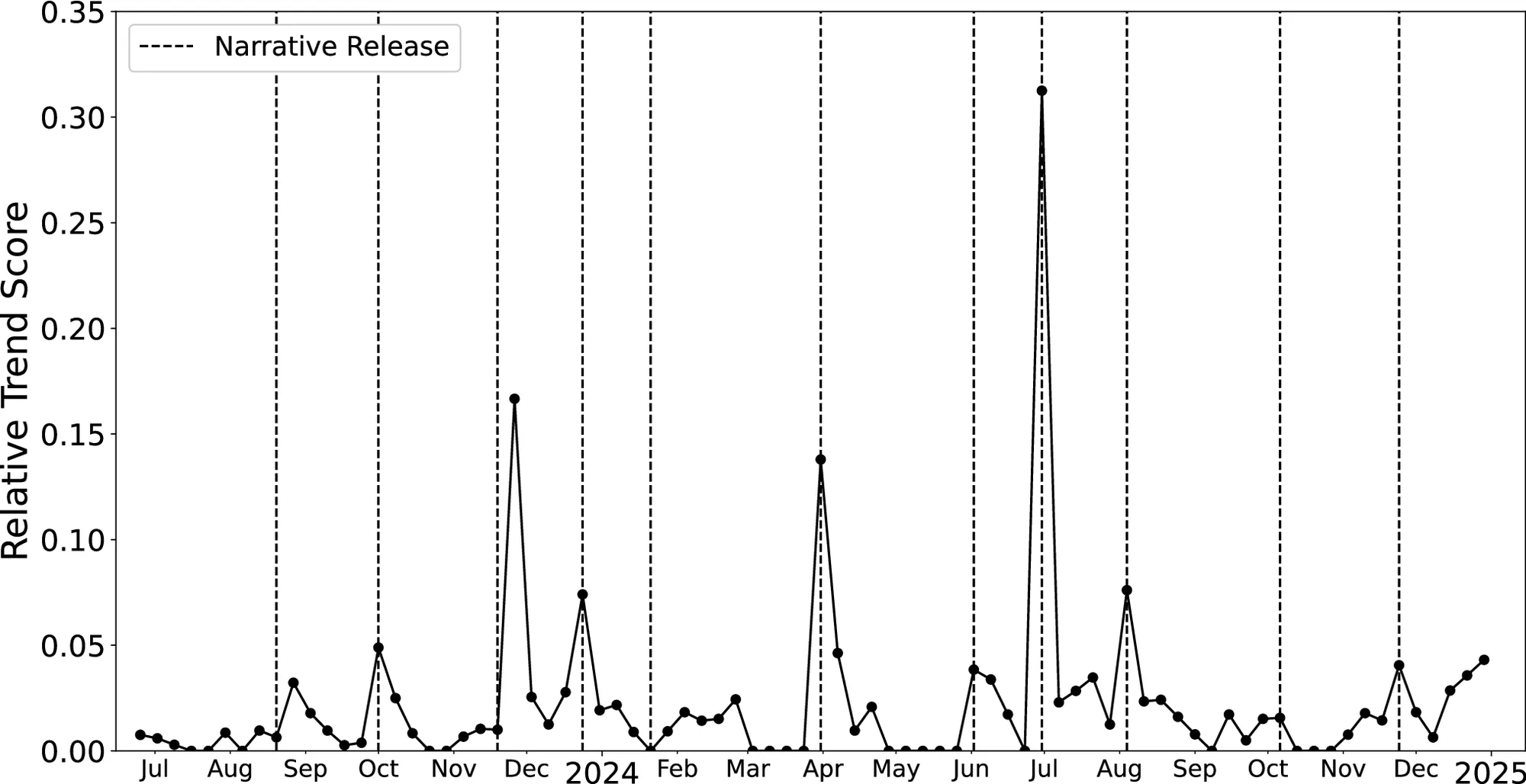
Weekly Google Search Trends score for searches including “Zelenskyy” and one of the Storm-1516 narrative keywords, relative to searches containing “Zelenskyy,” and timing of Storm-1516 narrative releases, July 1, 2023 to Dec 31, 2024.

To evaluate whether the introduction of these false narratives affected behavior beyond social media posts, we also measured search behavior. Users who encountered these narratives and wanted to evaluate their veracity could, for instance, turn to Google Search for more context. Fig. 4 presents the weekly relative search rank for searches containing both narrative-relevant keywords and Zelenskyy, compared with searches only containing Zelenskyy. As in previous figures, the vertical dotted lines indicate the weeks in which the Storm-1516 narratives first appeared on X. In almost all cases, the release of a narrative coincided with substantial spikes in the relative search rank for the associated keywords.

Following our regression approach to assessing message shares on X, we also measured how the Google search trend ratios changed in the week each narrative was introduced and each of the four subsequent weeks, relative to the 4-wk preintroduction period. The results of this analysis are presented in the third column of Table 1 (“Google Trend Share”). On average, across our 11 narratives, searches containing narrative-related keywords were substantively and statistically insignificant in the 4-wk preintroduction period, representing only 0.1% of the overall search score for Zelenskyy. In the week beginning the day the narratives were introduced, the relative score of Zelenskyy-related searches containing narrative keywords rose by 5.3 percentage points (*p* < 0.05). The elevated relative search score persisted in the following week, during which we observed a 2.5 percentage-point increase (*p* < 0.05) above the baseline. The score remained elevated in the second week after introduction, although this represented a much smaller 0.8 percentage point increase over the baseline (*p* < 0.05). By the third and fourth week following narrative introduction, Google Trends relative score seemed to remain slightly elevated, but at levels that were statistically indistinguishable from baseline.

## Discussion

Previous research has questioned the potential of FIMI to change audiences’ beliefs and behaviors (8, 9). These analyses answer a narrow but important question: how do the beliefs or behaviors of those who received a message from an influence campaign compare to those of similarly situated users who do not directly receive such a message. Here, we have argued that such comparisons may not be the only or most appropriate measure of success or failure of FIMI. Research has identified agenda setting as an alternate goal of some FIMI campaigns (10). This analysis of an ongoing Russian influence campaign shows that not only has Russian influence significantly affected discourse addressing the Ukrainian President and First Lady in ways that benefit Russian goals, but also that the campaign’s impact is consistent and persistent over time.

Agenda setting operates through several interconnected processes. First-level agenda setting describes how the repetition of media coverage affects which issues are prioritized in public discourse (11). By selecting which topics to report or omit, media organizations can shape which topics become prominent in public discussion (12). Second-level agenda setting concerns which attributes of an issue are emphasized (30). Research examining the effects of media agenda setting on the public has established that media framing can influence the salience of specific issues (31) and, through second-level agenda setting, the salience of specific issue attributes (32).

Our research demonstrates the effectiveness of a particular FIMI intervention that engaged in agenda setting to shift the salience of selected issue attributes. The successful use of agenda setting by this sort of actor has not been widely recognized. Moreover, we have demonstrated that the specific strategy they employ—narrative laundering—can successfully influence how an issue is discussed in public discourse. Although the effect of each individual intervention was short-lived, the campaign created a series of related narratives and consistently pushed them out over time. In this manner, the campaign maintained a high level of discourse around its chosen agenda. Here we might apply the metaphor of a doctor administering medicine to a patient, with each scheduled dose altering normal organic processes and each scheduled dose necessary to maintain that altered state.

Over the entire 18-mo period of our study, the baseline share of the discussion around Zelensky that involved accusations of corruption was 10.2 per 100 messages. In the 11 wk where Storm-1516 intervened in the conversation, that rate rose to 17 allegations per 100 messages. This 70% increase in the share of the discourse taken up by corruption was fleeting in each particular instance, but these 11 interventions together meant that, on average, the share of the conversation devoted to allegations of corruption in the full 78-wk period was at about 10% higher than it would otherwise have been.

There are many ways President Zelenskyy might be framed; it is in Russia’s interest that he be portrayed not as a hero but as a villain. Narrative laundering enables the Russian FIMI to describe Zelenskyy as corrupt and self-serving, a leader focused on lining his own pockets at the expense of his country. While there are significant empirical challenges in establishing causal relationships between these narrative-laundering efforts and attitudinal change in Russia’s target audience, it is the case that influential Republican policymakers have themselves promoted false Storm-1516 narratives. For example, Congresswoman Marjory Taylor Green linked to the Storm-1516 “yacht” narrative on her X account soon after it was introduced, saying “Anyone who votes to fund Ukraine is funding the most corrupt money scheme of any foreign war in our country’s history” (22). Indeed, it is also true that pro-Ukrainian attitudes have fallen among Republican-leaning Americans over the past 2 y, but not among Democratic Americans (33).

This research does face certain limitations worth noting. First, we may not have fully accounted for the possibility that some posts promoting Storm-1516 narratives in the period of study were made by inauthentic accounts, perhaps even bot accounts deployed by the campaign. Estimates of the prevalence of inauthentic accounts on X vary (34), but they are known to be deployed in FIMI operations. It is possible inauthentic accounts helped amplify the Zelenskyy narratives on X, but no reporting to date has shown that to be the case. In particular, Viginum’s detailed analysis of how Storm-1516 spread through social media did not identify bot amplification. As a robustness check, we repeated both social-media analyses after removing all posts from users in the bottom two deciles of follower counts, as low follower counts have been shown to be a robust (if imperfect) predictor in social-bot detection (35). The results (presented in *SI Appendix*, section S4) are similar to or larger than our full-sample results. Further, the parallel results we identified between narrative discussion on X and narrative search behavior on Google suggest that genuine users are engaging with the discourse. Just as inauthentic narrative amplification might cause us to overestimate the impact of the Storm-1516 campaign, a second possible limitation might cause us to underestimate it: users may discuss a narrative in terms other than the keywords included in our measures. Conversations about a Zelenskyy narrative that did not contain our selected keywords would not be captured in our estimates, potentially understating the true share of discourse around these narratives.

Our LLM-based measure of the effect of Storm-1516 on the Zelensky corruption metanarrative is also subject to measurement limitations. Although the classifier did align quite well with the human coding, it was not perfect. To the extent that the remaining classification error is random, its influence should be reduced by aggregating over large numbers of posts within each week. However, if the LLM coding was particularly good (bad) at recognizing corruption messages aligned with Storm-1516 narratives, it could over- (under-)estimate the impact of the Storm-1516 intervention on discussion of the corruption metanarrative.

This work also demonstrates the weakness of some countermeasures, such as fact-checking by traditional news outlets. The existence of and tactics used by the Storm-1516 campaign has been widely recognized since late 2023 (36). Nearly every narrative in our study was subjected to fact-checking, often quite rapidly (See *SI Appendix*, section S1 for details). Nevertheless, fact-checking of the corruption metanarrative accounts for less than 0.4% of the full discourse around Zelenskyy, outnumbered by posts supporting the metanarrative by more than 27-to-1.

The presence of 11 closely related false narratives allowed us to investigate the impact of this sort of narrative-laundering FIMI intervention on the mix of conversations around Zelenskyy quantitatively. But the Storm-1516 campaign was substantially broader, both in how it targeted Zelenskyy and in how it targeted other elements of political discourse on topics relevant to Russia. These 11 false narratives represented only half the false narratives targeting Zelenskyy in this period, and only 17% of the (known) Storm-1516 output in this period. Over this 18-mo period, the operation produced, on average, approximately one false narrative per week. If the remaining narratives generated effects comparable in magnitude and persistence to those we measured here, the campaign had a sustained influence over roughly 7 to 10% of discourse pertaining to its targeted topics, both in terms of posts on X and in Google Search behavior, throughout this period.

## Supplementary Material

Appendix 01 (PDF)

## Data Availability

Excel/CSV data will be deposited in Narrative Laundering replication package (10.5281/zenodo.21681897) (27).

## Footnotes

^*^Fig. 2 shows a slight delay for online discussion of the February 2024 narrative relative to other narratives. Storm-1516 typically uses X to layer and integrate narratives that were first introduced elsewhere (i.e., off the platform); this narrative (re: the purchase of a luxury apartment in Dubai) was uniquely first placed on X with a post from a fake journalist. There is typically a period of several days or weeks between placement and integration. Given this narrative was first placed on X, the timing of its spread on X differs from the others as we measure from a narrative’s first appearance on X.

^†^Fig. 3 shows several spikes in corruption related discourse unrelated to Storm-1516 narratives. Each appears to be the result of real-world events that reasonably result in such an increase. The October 2023 spike, for instance, followed a Time Magazine cover story which discussed the Zelenskyy regime’s efforts to fight corruption while the July 2024 spike follows a visit by Zelenskyy to Washington.
