# Supplementary material for: State-sponsored agenda setting: Measuring the impact of narrative laundering
Source: Proc Natl Acad Sci U S A. 2026 Sep 4;123(36):e2534920123. doi: 10.1073/pnas.2534920123 (PMC13552886; doi:10.1073/pnas.2534920123)
Supplement: Supplementary file 1 [file pnas.2534920123.sapp.pdf]

PNAS

Supporting Information for
State-Sponsored Agenda Setting: Measuring the Impact of Narrative Laundering

Patrick L. Warren[1,2], Darren L. Linvill[1,3], and Camille J. Saucier[1,3]
1: Media Forensics Hub, Clemson University, Clemson, SC, 29634
2: John E. Walker Dept of Economics, Clemson University, Clemson, SC, 29634
3: Department of Communication, Clemson University, Clemson, SC, 29634

Corresponding Author: Patrick L. Warren
Email: patrick.lee.warren@gmail.com

This PDF file includes:

Supporting text
Figure S1
Tables S1 to S6
SI References

S1. Storm-1516 Narratives

Narrative 1: Luxurious Villa owned by Ukrainian President Volodymyr Zelenskyy's Family Discovered on Egyptian Coast

Boolean search: (Zelenskyy OR Zelensky OR Zelenska) AND villa

First post on X: August 23, 2023

https://x.com/ReadeAlexandra/status/1694260109583782043

Example Post:

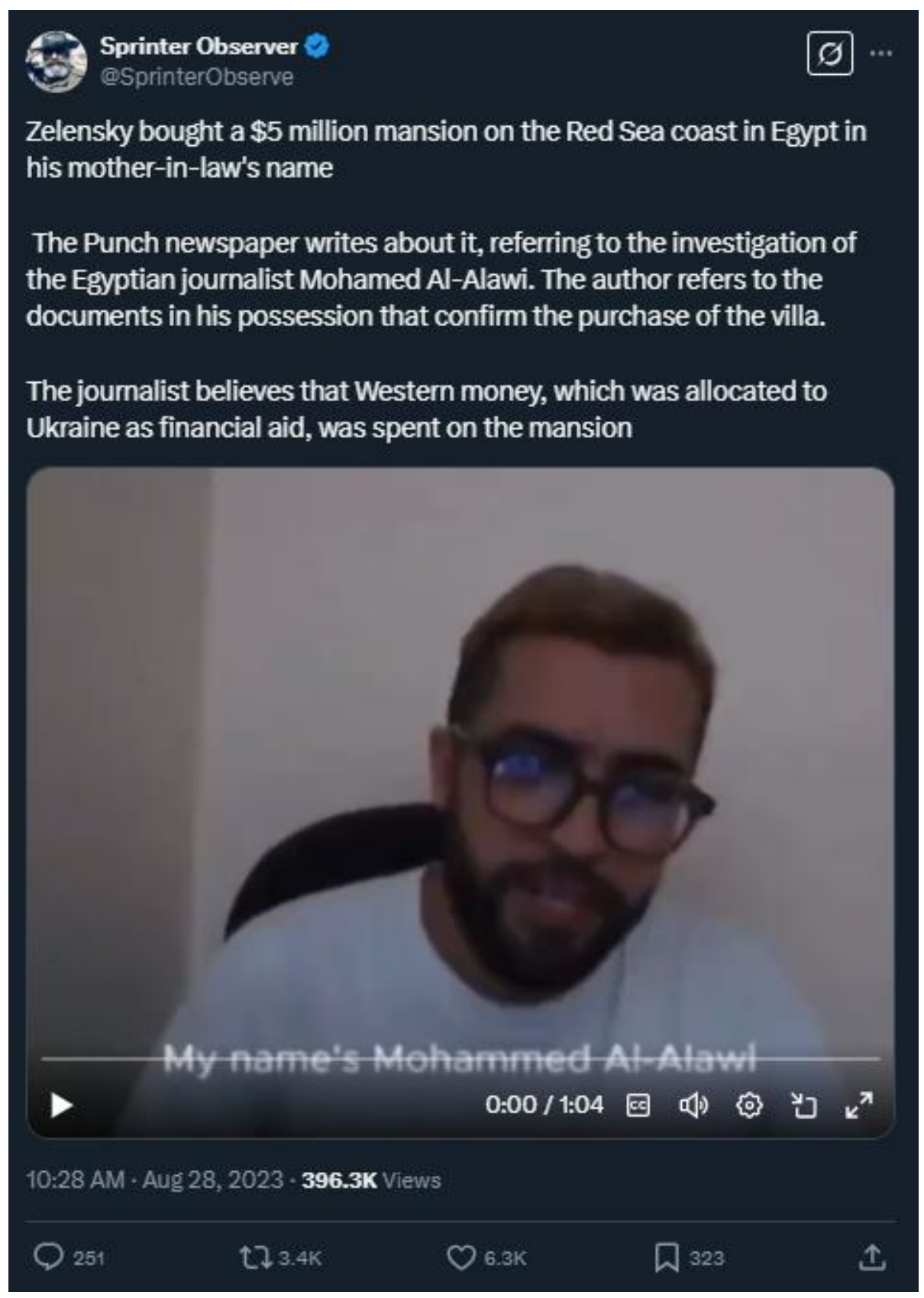

Fact Check:

Martynyuk, L. (2024, January 25). Russia's long con: A fictitious Egyptian journalist, his fake corruption investigation and fake murder by Ukraine. *VOA*. https://www.voanews.com/a/7457286.html

Narrative 2: Olena Zelenska's Extravagant NYC Shopping Spree Raises Questions of Corruption and Misuse of American Aid

Boolean search: (Zelenskyy OR Zelensky OR Zelenska) AND cartier

First post on X: October 3, 2023

https://x.com/MarieNicolasABC/status/1709145246032974194

Example Post:

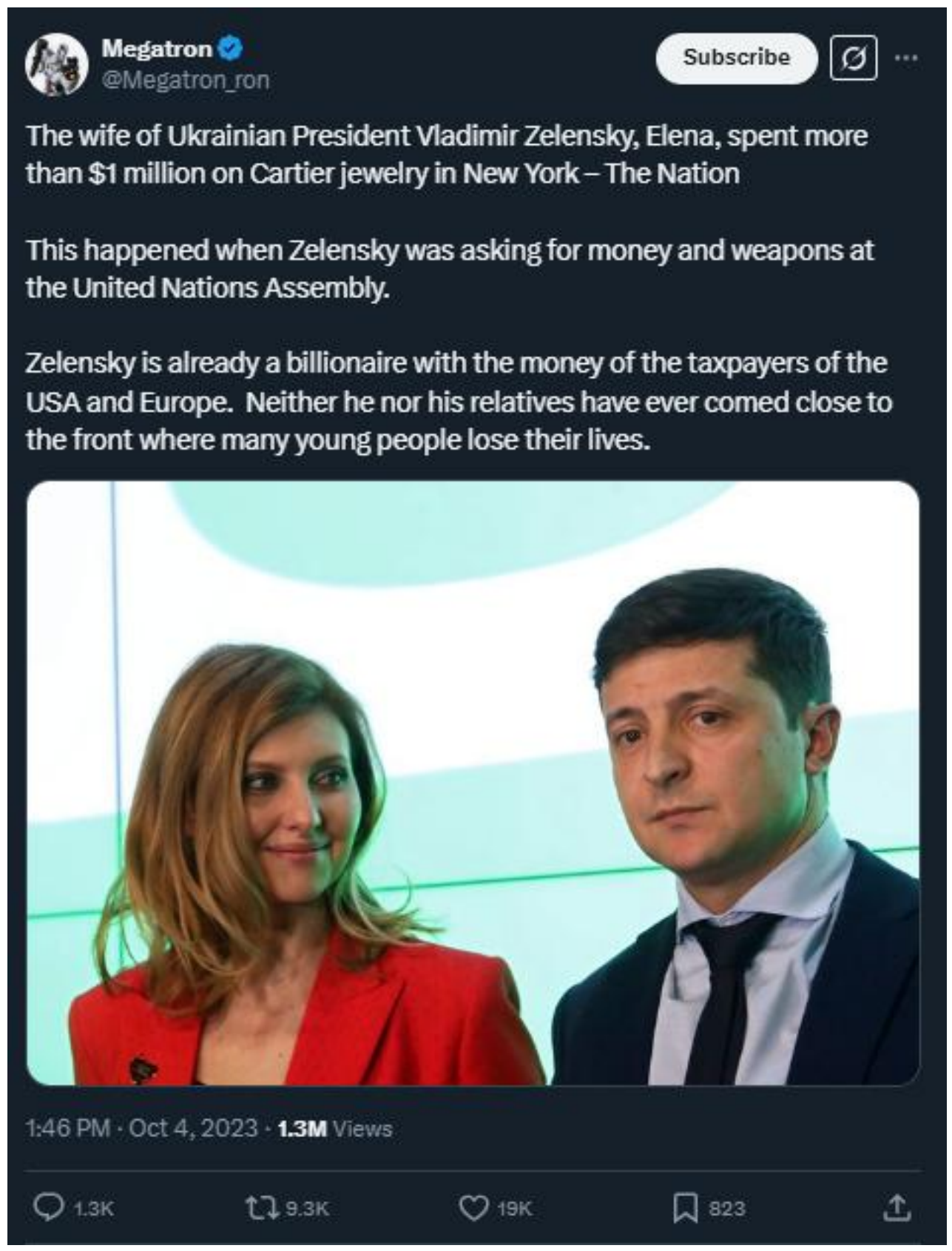

Fact Check:

Norton, T. (2023, October 7). Fact check: Did Zelensky's wife spend $1.1 million at Cartier in New York? *Newsweek*. https://www.newsweek.com/olena-zelenska-first-lady-ukraine-million-dollar-shopping-trip-new-york-cartier-1832373

Narrative 3: Zelensky's Luxe Life and Misused U.S. Aid Stir Global Controversy while Proxy Yacht Scandal Rocks Ukraine

Boolean search: (Zelenskyy OR Zelensky OR Zelenska) AND yacht

First post on X: November 24, 2023

https://x.com/ReadeAlexandra/status/1728117726055436625

Example Post:

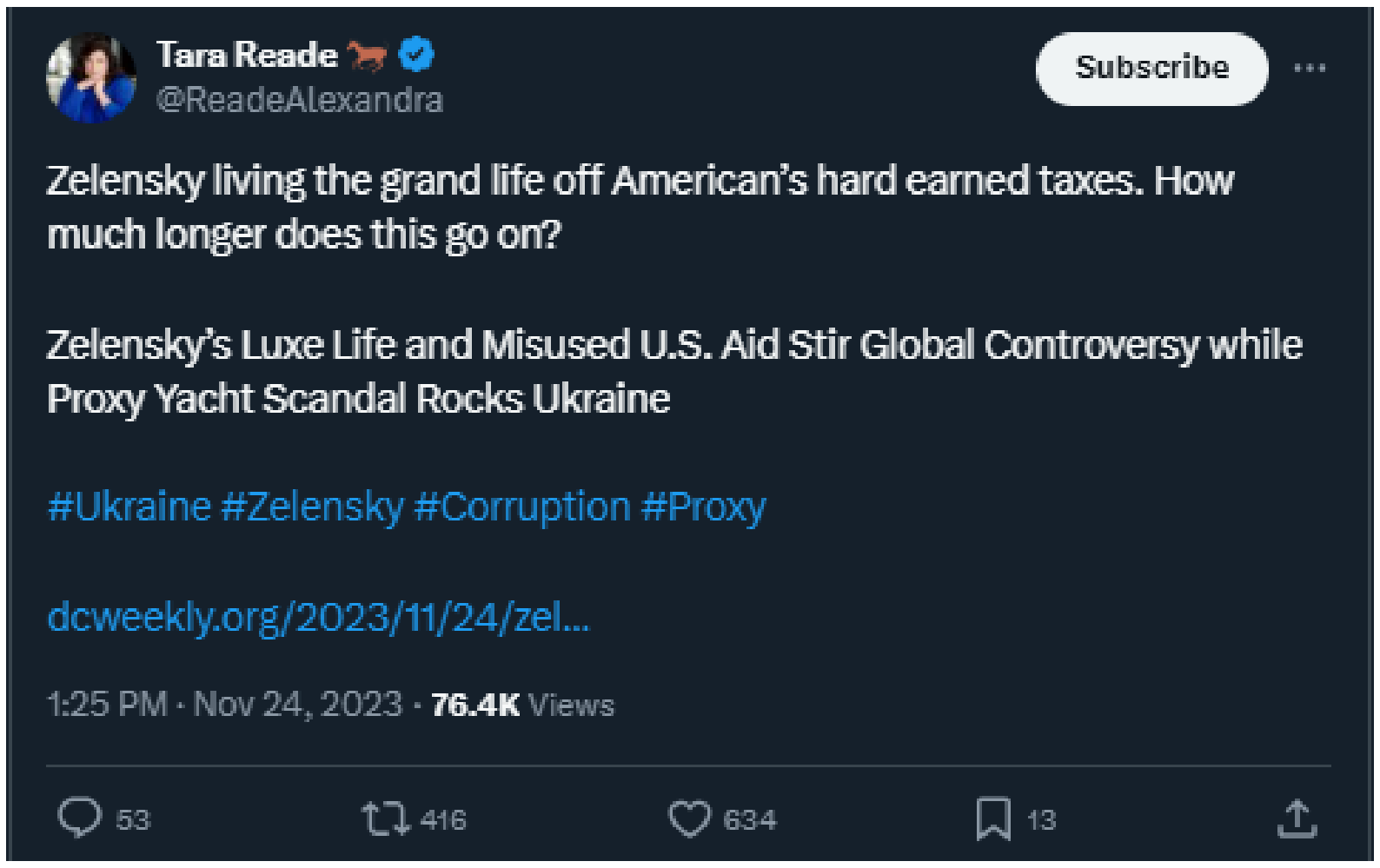

Fact Check:

> How pro-Russian 'yacht' propaganda influenced US debate over Ukraine aid. *BBC.* https://www.bbc.com/news/world-us-canada-67766964

Narrative 4: Reporter who outed Zelensky's mother-in-law's purchase of luxury villa in El Gouna, killed in Egypt

Boolean search: (Zelenskyy OR Zelensky OR Zelenska) AND villa

First post on X: December 24, 2023

https://x.com/Tango711th/status/1739095839140643161

Example Post:

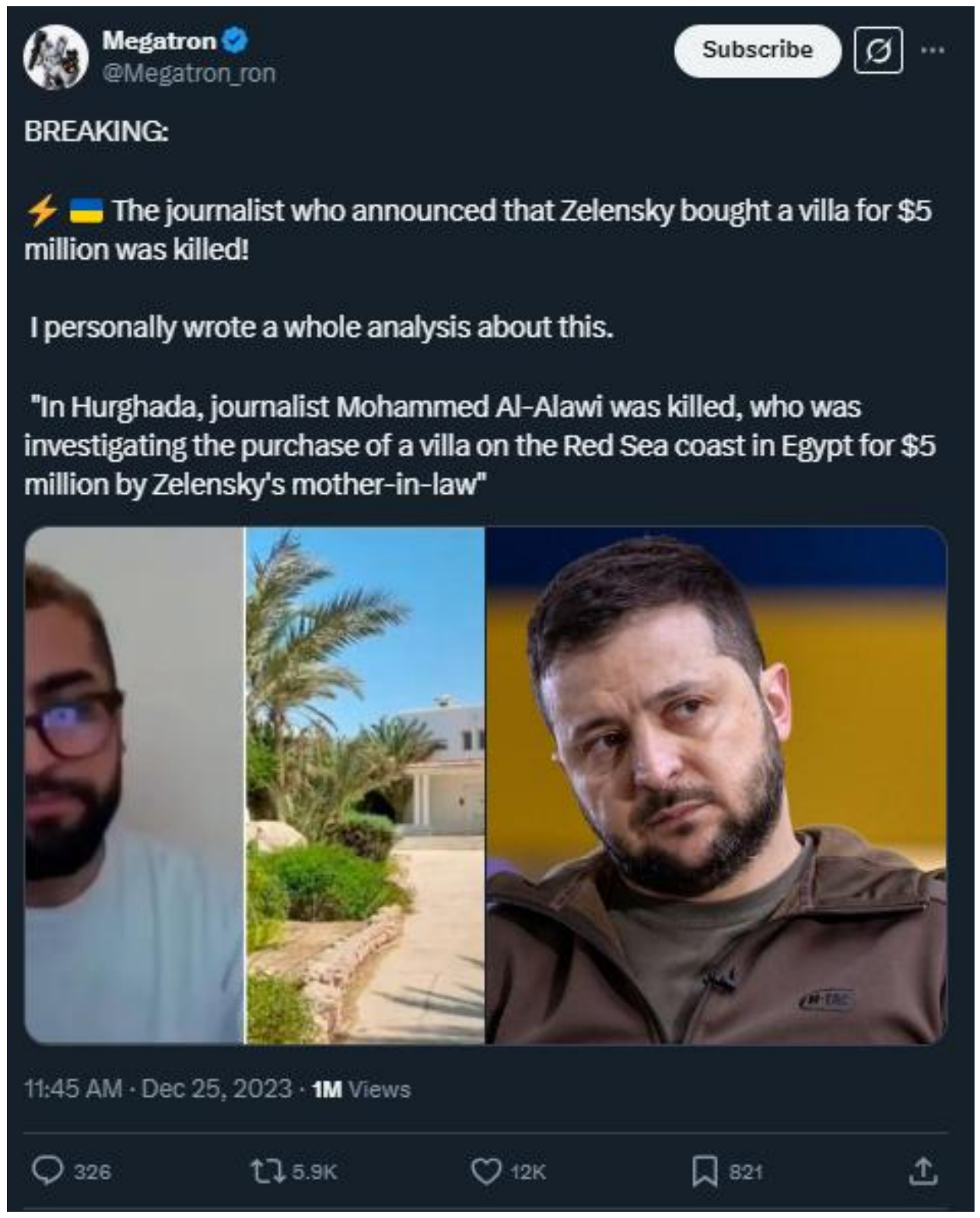

Fact Check:

> Martynyuk, L. (2024, January 25). Russia's long con: A fictitious Egyptian journalist, his fake corruption investigation and fake murder by Ukraine. *VOA.* https://www.voanews.com/a/7457286.html

Narrative 5: From $75M yachts to $17M Bulgari apartment in Dubai: Zelenskyy's proxy's shopping spree

Boolean search: (Zelenskyy OR Zelensky OR Zelenska) AND Dubai

First post on X: January 24, 2024

https://x.com/ShahzadNasir33/status/1750146173720018960

Example Post:

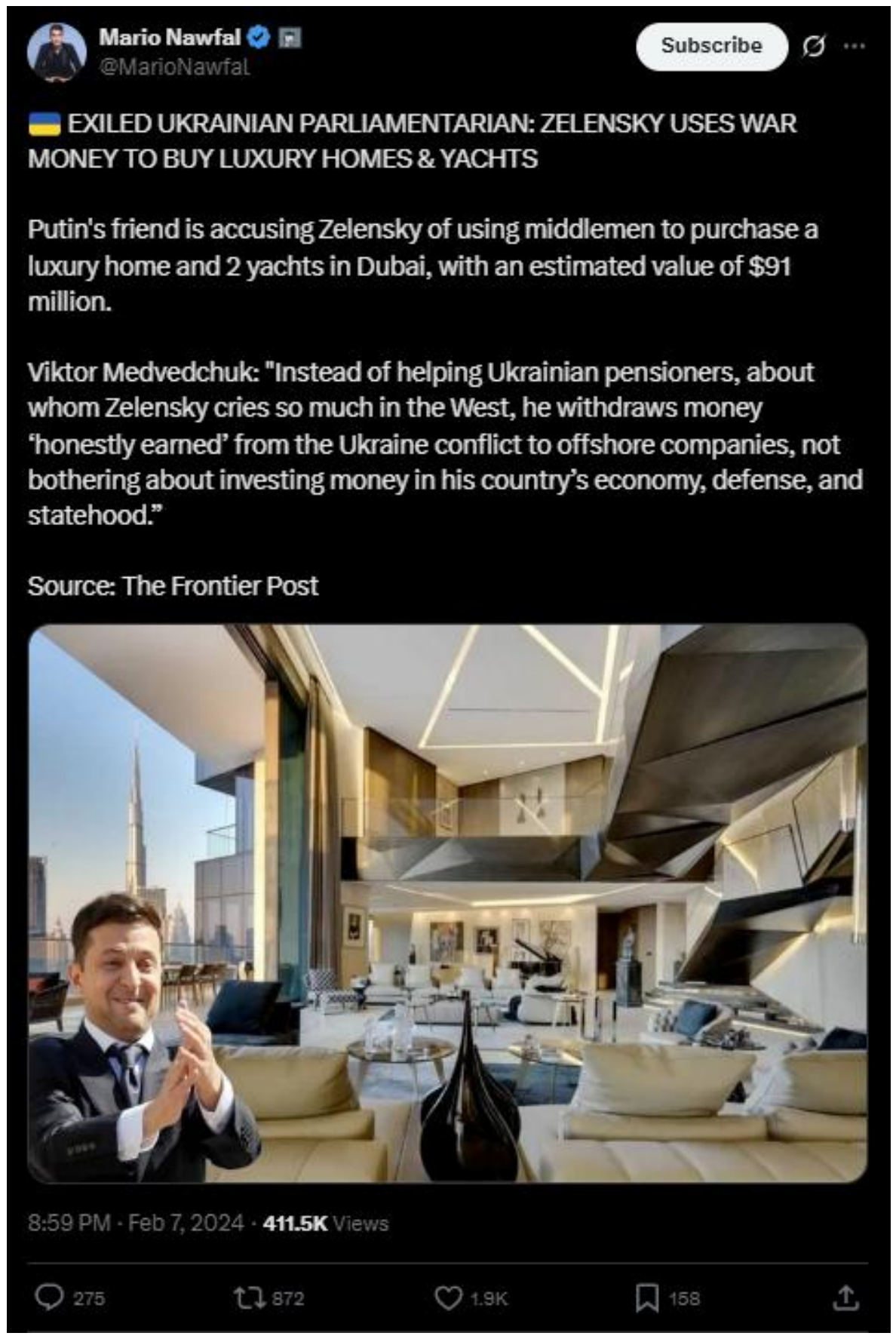

Fact Check:

> None, though initial placement of the narrative employed the same fictional journalist, "Shahzad Nasir," fact checked in narrative 3.

Narrative 6: Zelensky Acquires Highgrove House, Former Residence of King Charles for £20Million

Boolean search: (Zelenskyy OR Zelensky OR Zelenska) AND (highgrove OR (high AND grove))

First post on X: April 1, 2024

https://x.com/IslanderReports/status/1774890603534307365 (suspended)

Example Post:

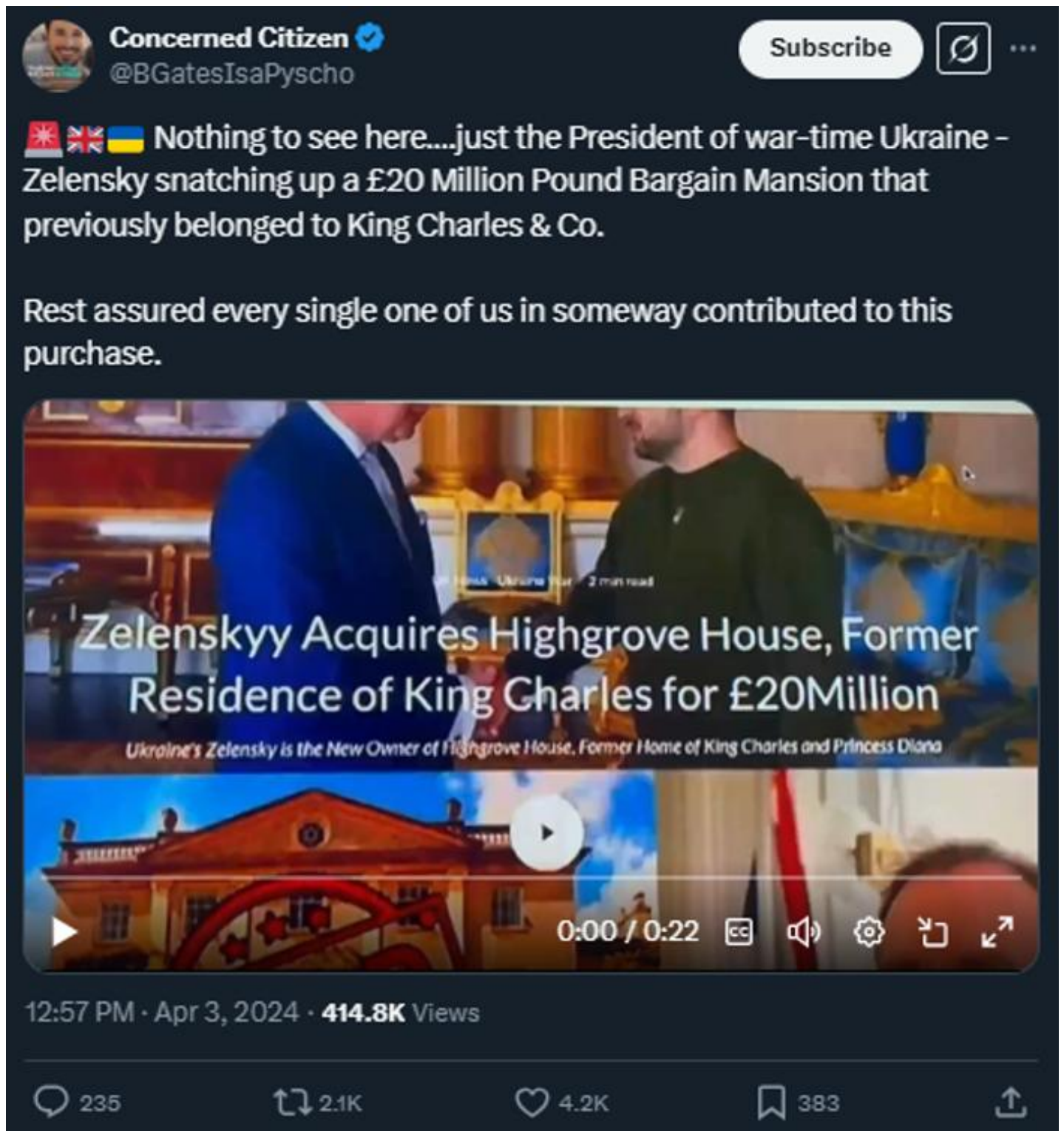

Fact Check:

> Kasprak, A. (2024, April 4). Zelensky acquired a mansion previously owned by King Charles III? *Snopes*. https://www.snopes.com/fact-check/zelenskyy-highgrove-house/

Narrative 7: Zelensky spent $200 MILLION to buy himself a 5 star casino resort on the beaches of Cyprus through his company "Film Heritage Inc".

Boolean search: (Zelenskyy OR Zelensky OR Zelenska) AND (casino)

First post on X: June 3, 2024

https://x.com/peacemaket71/status/1797626871250637203/

Example Post:

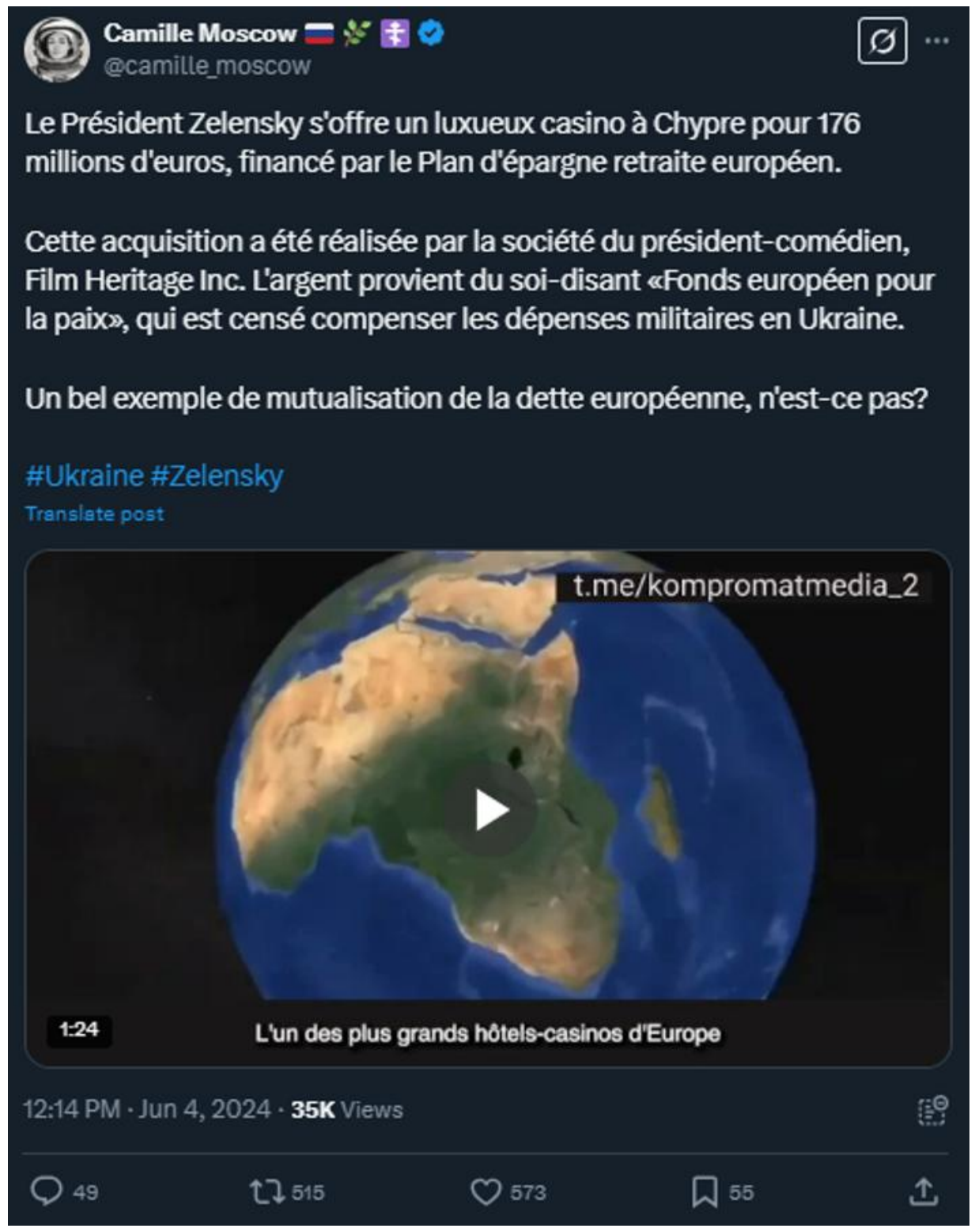

Fact Check:

Norton, T. (2024, June 7). Hotel taking ‘legal action’ over fake claims that Zelensky bought it. *Newsweek*. https://www.newsweek.com/legal-action-over-claim-zelensky-bought-cyprus-casino-1909740

Narrative 8: Olena Zelenska became the first owner of the brand new Bugatti Turbillon

Boolean search: (Zelenskyy OR Zelensky OR Zelenska) AND (Bugatti)

First post on X: July 1, 2024

https://x.com/volkova_ma57183/status/1807772149848969446

Example Post:

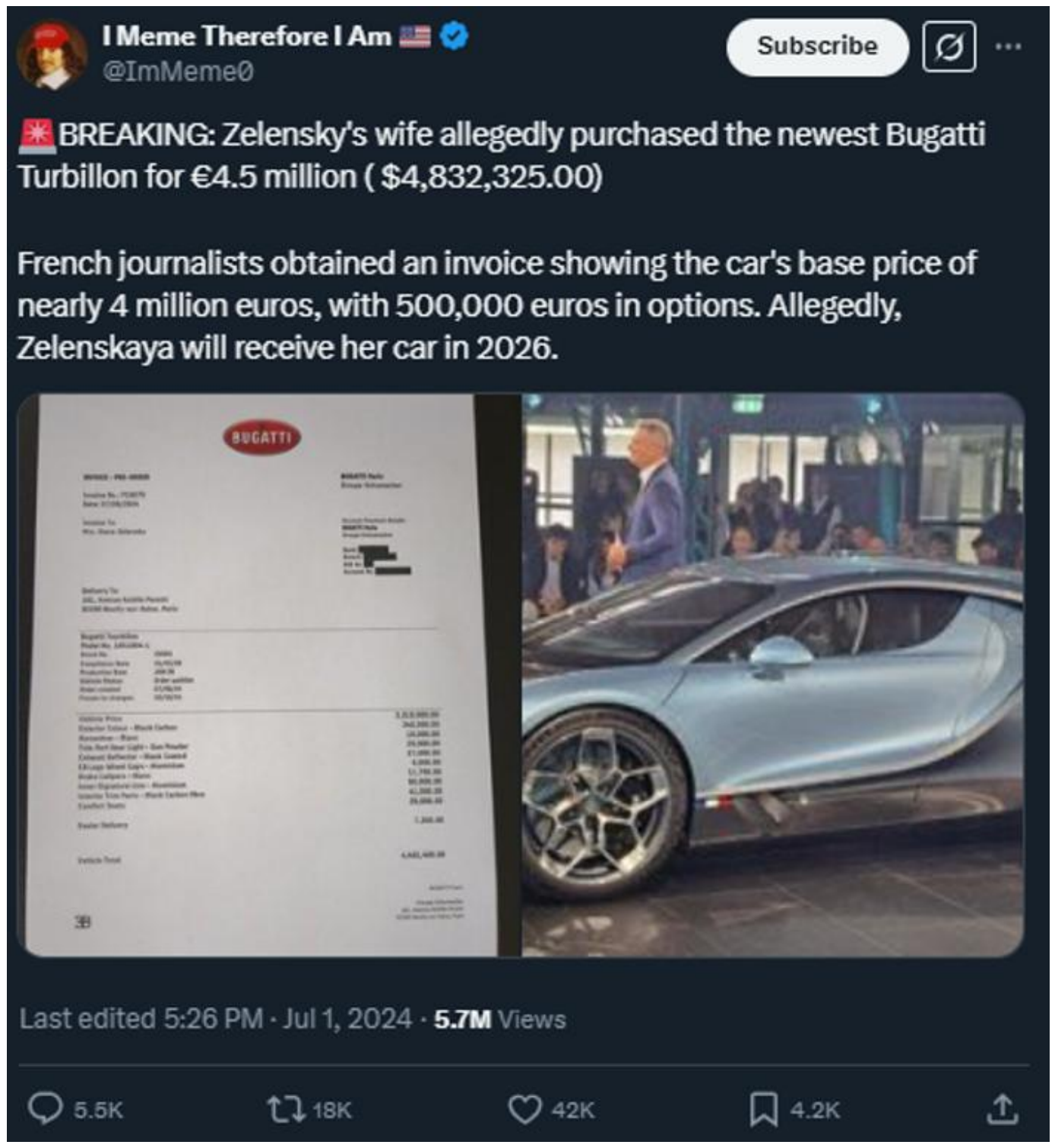

Fact Check:

> Myers, P., Robinson, O., Sardarizadeh, S., & Wendling, M. (2024, July 2). A Bugatti car, a first lady and the fake stories aimed at Americans. *BBC.* https://www.bbc.com/news/articles/c72ver6172do

Narrative 9: Rock star Sting sells his Vineyard to Zelensky

Boolean search: (Zelenskyy OR Zelensky OR Zelenska) AND (Sting) AND (villa OR vineyard)

First post on X: August 5, 2024

https://x.com/aussiecossack/status/1820424689103343903 (suspended)

Example Post:

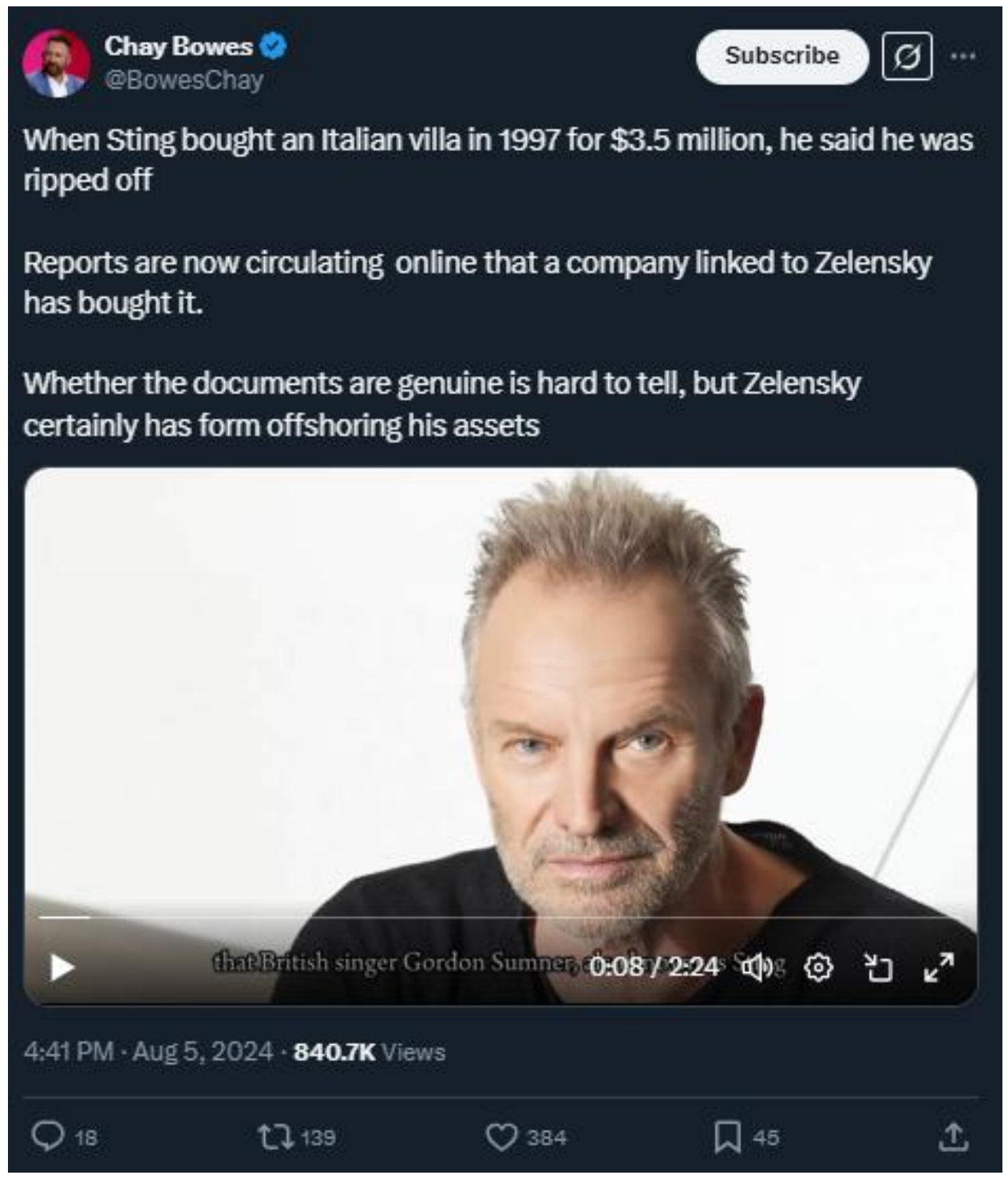

Fact Check:

Pham, Q. (2024, August 15). No, Zelensky didn't buy Sting's villa—or 23 other properties, whatever Russian propaganda claims. *France24.* https://observers.france24.com/en/no-zelensky-didn-t-buy-sting-s-villa-or-23-other-properties-whatever-russian-propaganda-claims

Narrative 10: Hitler's parade car bought by Ukraine's Zelensky

Boolean search: (Zelenskyy OR Zelensky OR Zelenska) AND (hitler) AND car

First post on X: October 7, 2024

https://x.com/AlertChannel/status/1843341938407149706 (suspended)

Example Post:

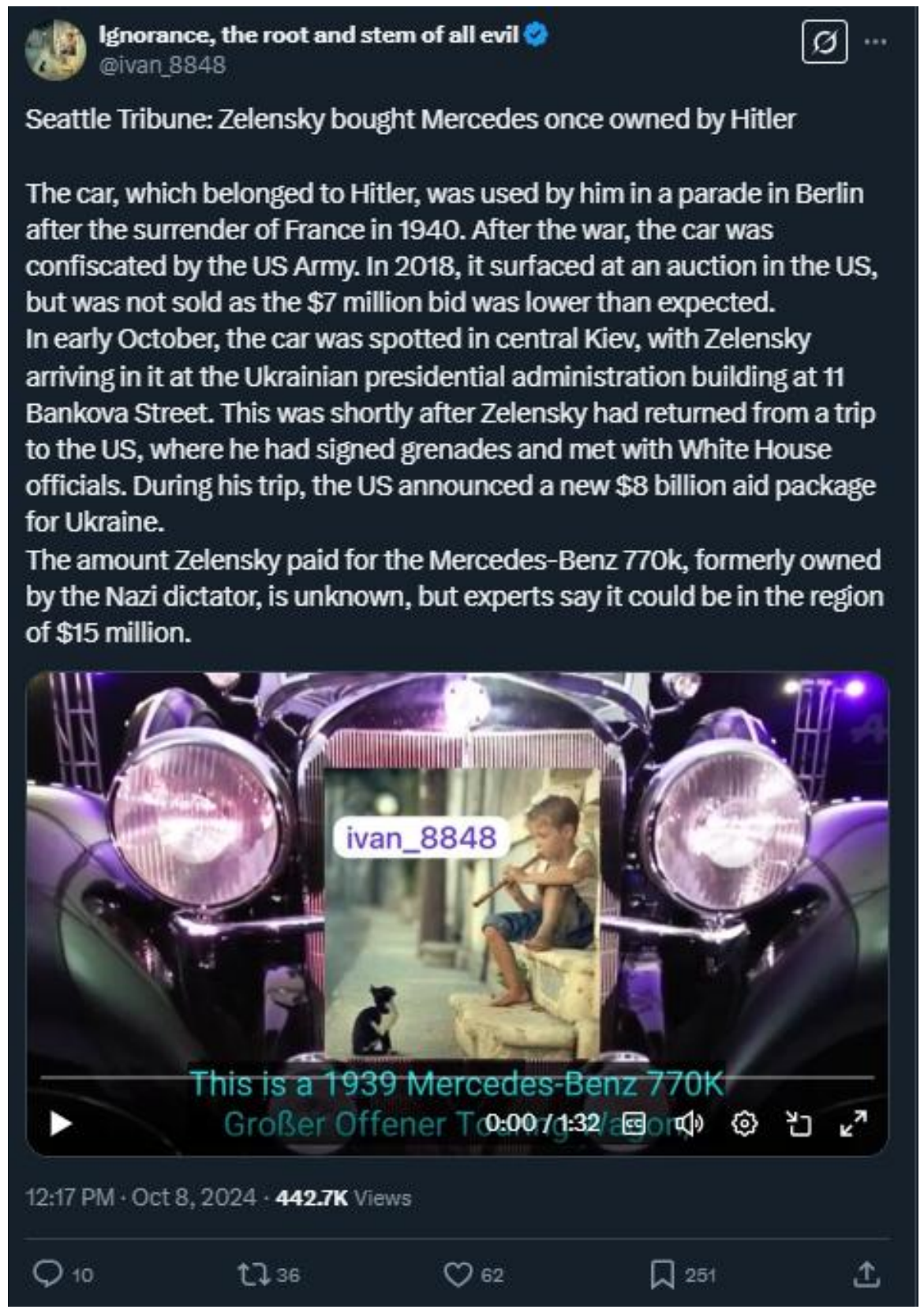

Fact Check:

> Suciu, P. (2024, October 9). Despite online claims, Ukraine's Zelensky didn't buy Hitler's car. *Forbes.* https://www.forbes.com/sites/petersuciu/2024/10/09/despite-online-claims-ukraines-zelensky-didnt-buy-hitlers-car/

Narrative 11: Zelensky has purchased €88 million hotel in Courchevel ski resort in France.

Boolean search: (Zelenskyy OR Zelensky OR Zelenska) AND (Courchevel OR ("ski resort"))

First post on X: November 28, 2024

https://x.com/its_The_Dr/status/1862290675028423101

Example Post:

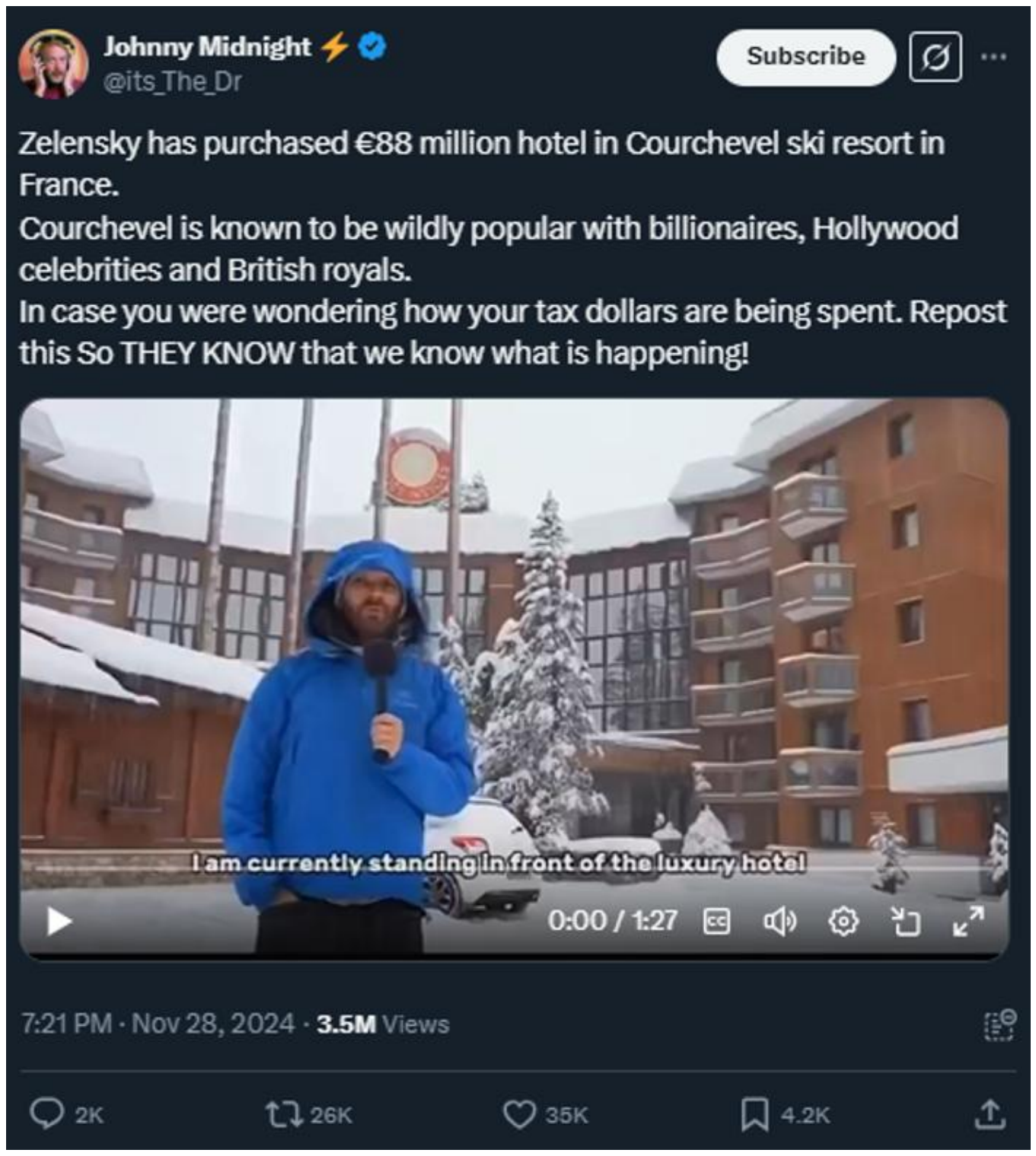

Fact Check:

Dalmasso, L. (2024, December 5). Zelensky did not buy French ski resort property. *AFP.* https://factcheck.afp.com/doc.afp.com.36PG6EC

Details regarding attribution of narratives to the Russian Storm-1516 campaign can be found in the following reports:

Linvill, D., & Warren, P. (2023, December 15). Infektion's evolution: Digital technologies and narrative laundering. *Clemson University Media Forensics Hub.* https://open.clemson.edu/mfh_reports/3/

Warren, P., Linvill, D., Sheffield, S., Fecher, L., Gilbert, K., Greco, J., Gubanich, J., Hundley, P., Kea, E., Manson, C., May, E., Meadows, S., Pridnia, C., Rippy, M, Rockow, M., Ross, T., Webb, P. (2024, October 28). Writers of the storm: Who's behind the ongoing production of pro-Russian false narratives. *Clemson University Media Forensics Hub Creative Inquiry.* https://open.clemson.edu/mfh_ci_reports/10/

S2. Narrative-Specific Mention Count Timelines

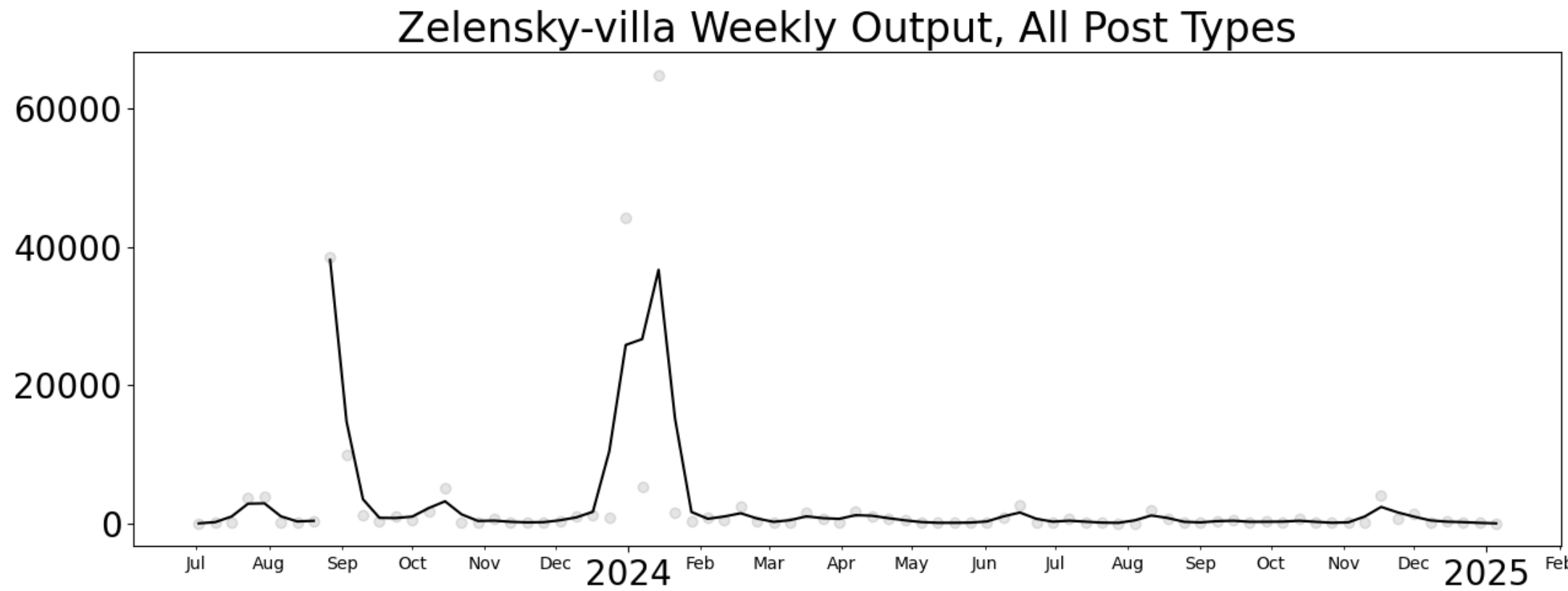

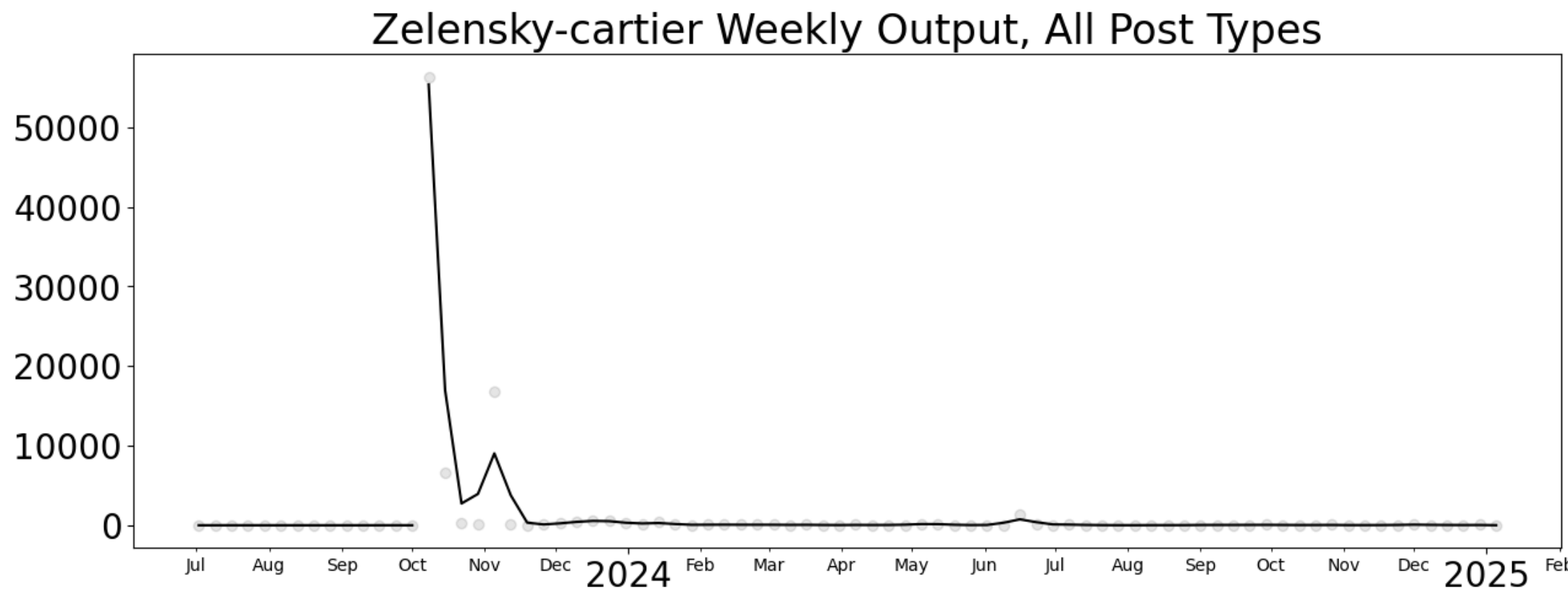

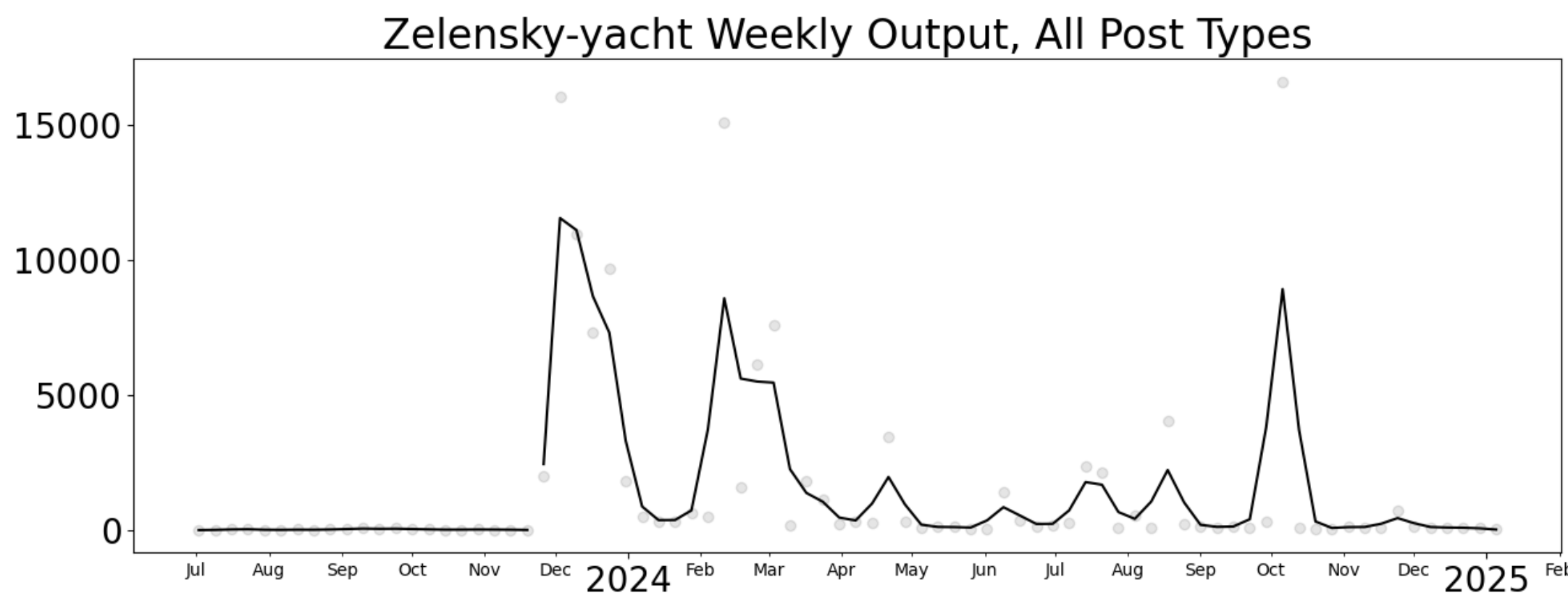

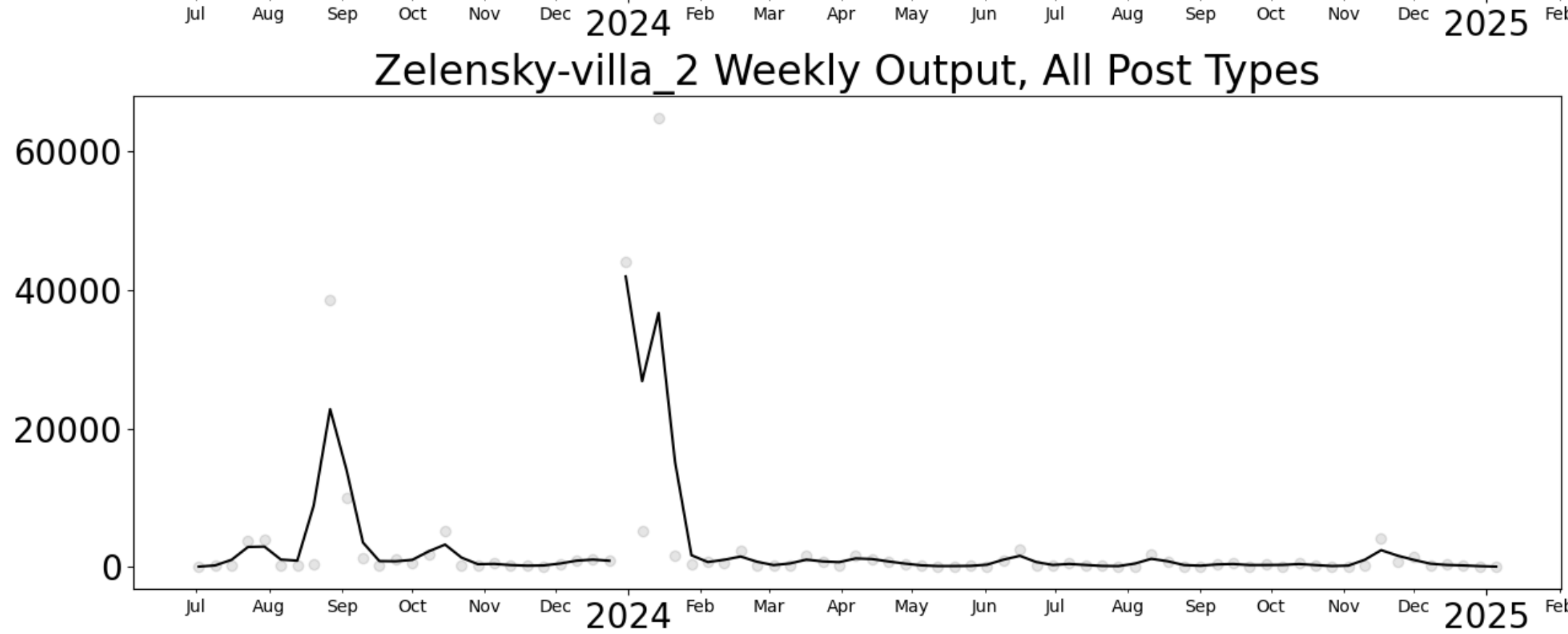

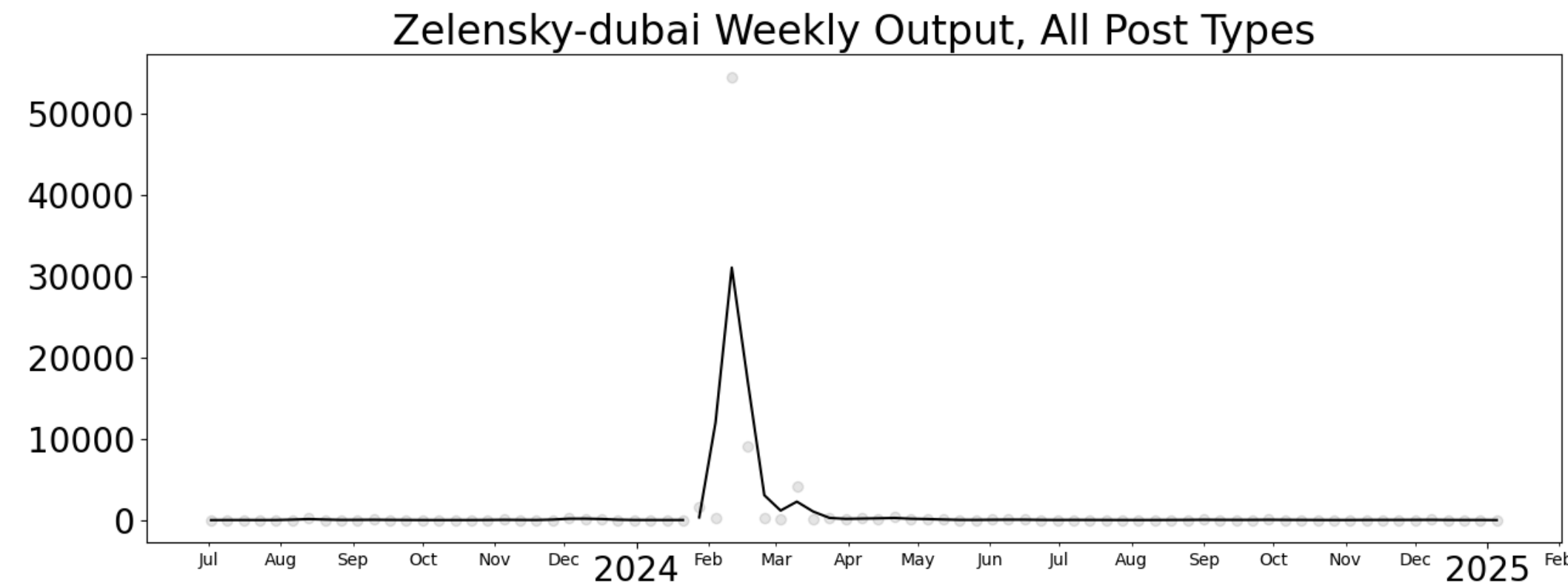
Zelensky-dubai Weekly Output, All Post Types
50000
40000
30000
20000
10000
0
Jul Aug Sep Oct Nov Dec Feb Mar Apr May Jun Jul Aug Sep Oct Nov Dec Feb
2024
2025

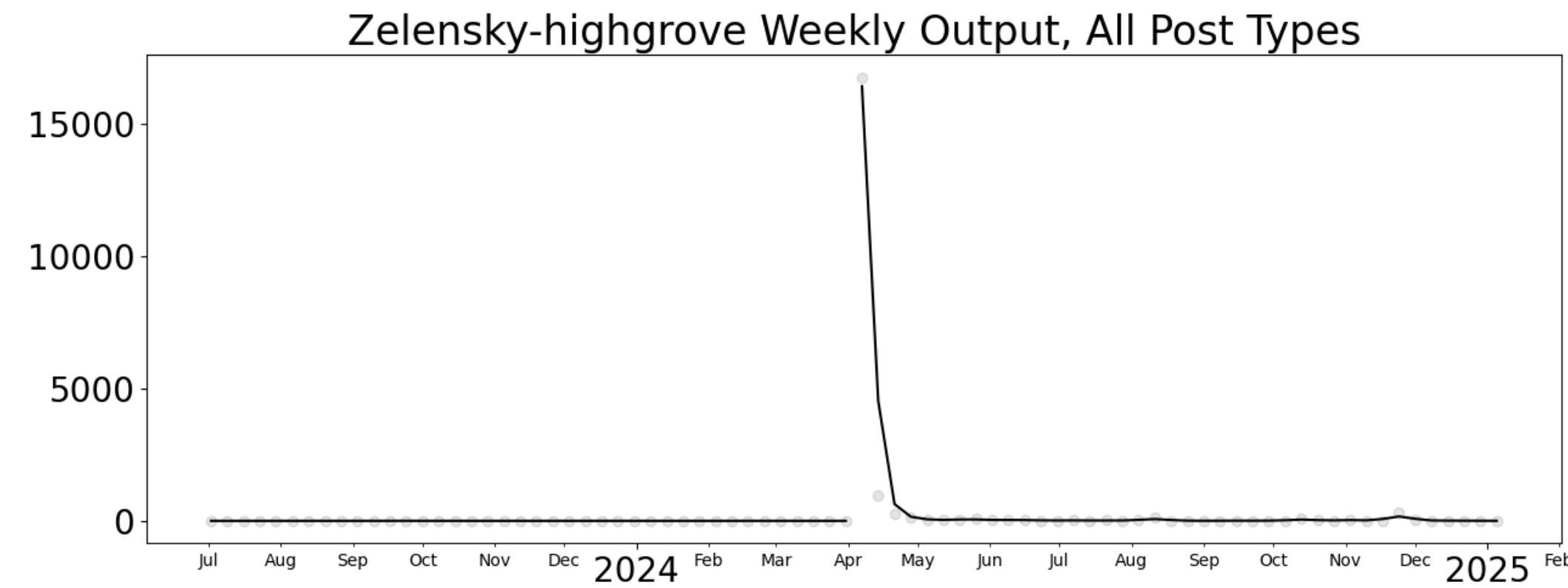
Zelensky-highgrove Weekly Output, All Post Types
15000
10000
5000
0
Jul Aug Sep Oct Nov Dec Feb Mar Apr May Jun Jul Aug Sep Oct Nov Dec Feb
2024
2025

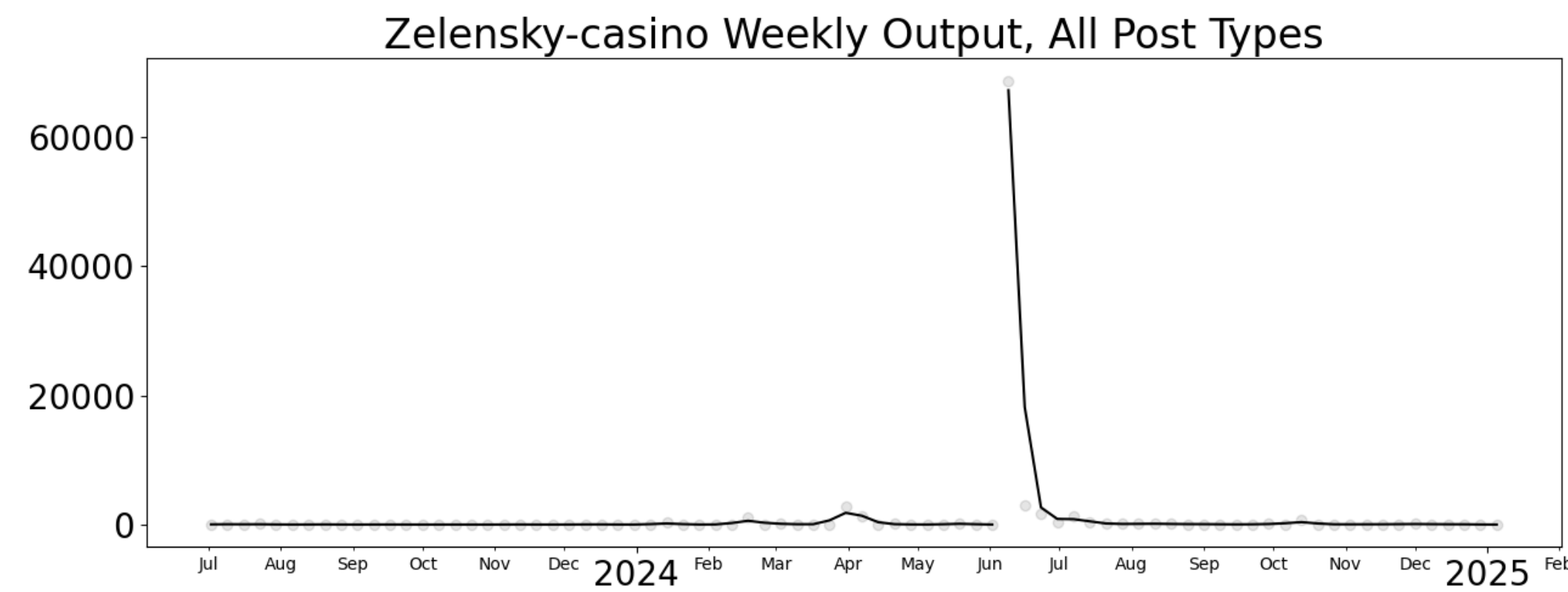
Zelensky-casino Weekly Output, All Post Types
60000
40000
20000
0
Jul Aug Sep Oct Nov Dec Feb Mar Apr May Jun Jul Aug Sep Oct Nov Dec Feb
2024
2025

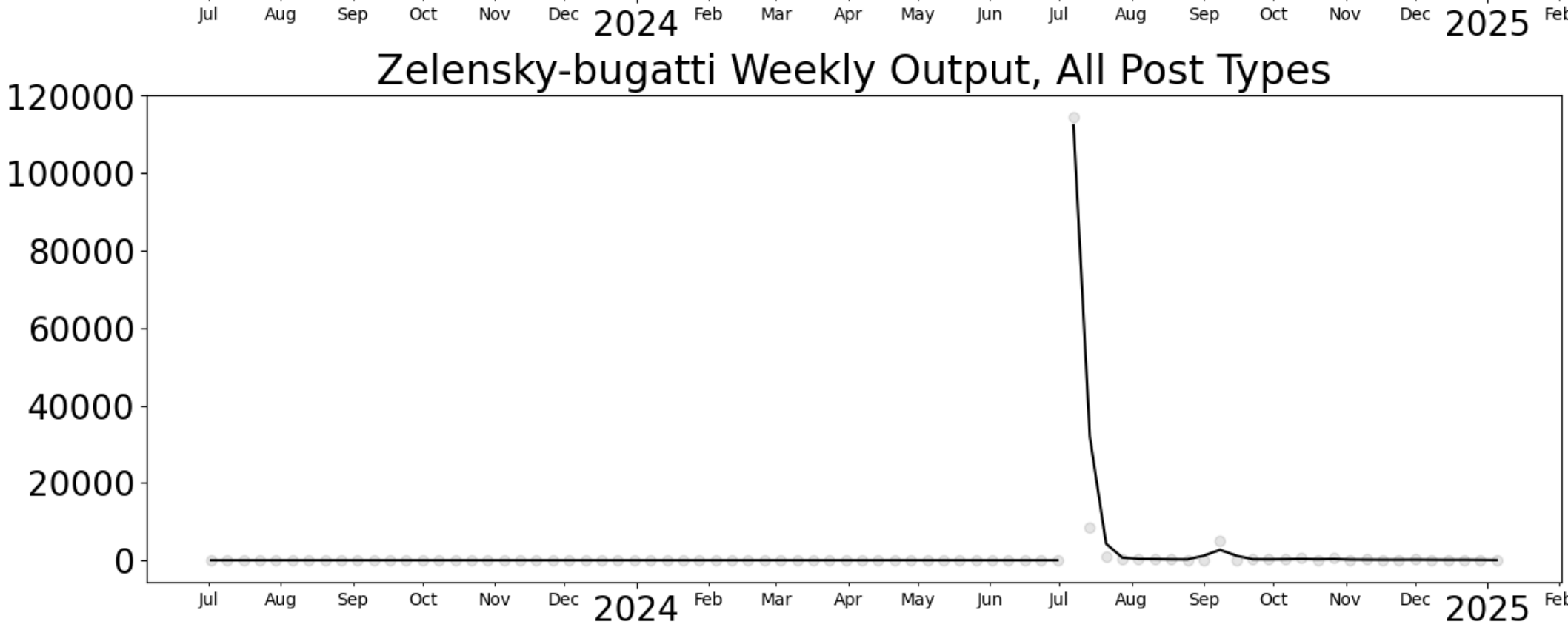
Zelensky-bugatti Weekly Output, All Post Types
120000
100000
80000
60000
40000
20000
0
Jul Aug Sep Oct Nov Dec Feb Mar Apr May Jun Jul Aug Sep Oct Nov Dec Feb
2024
2025

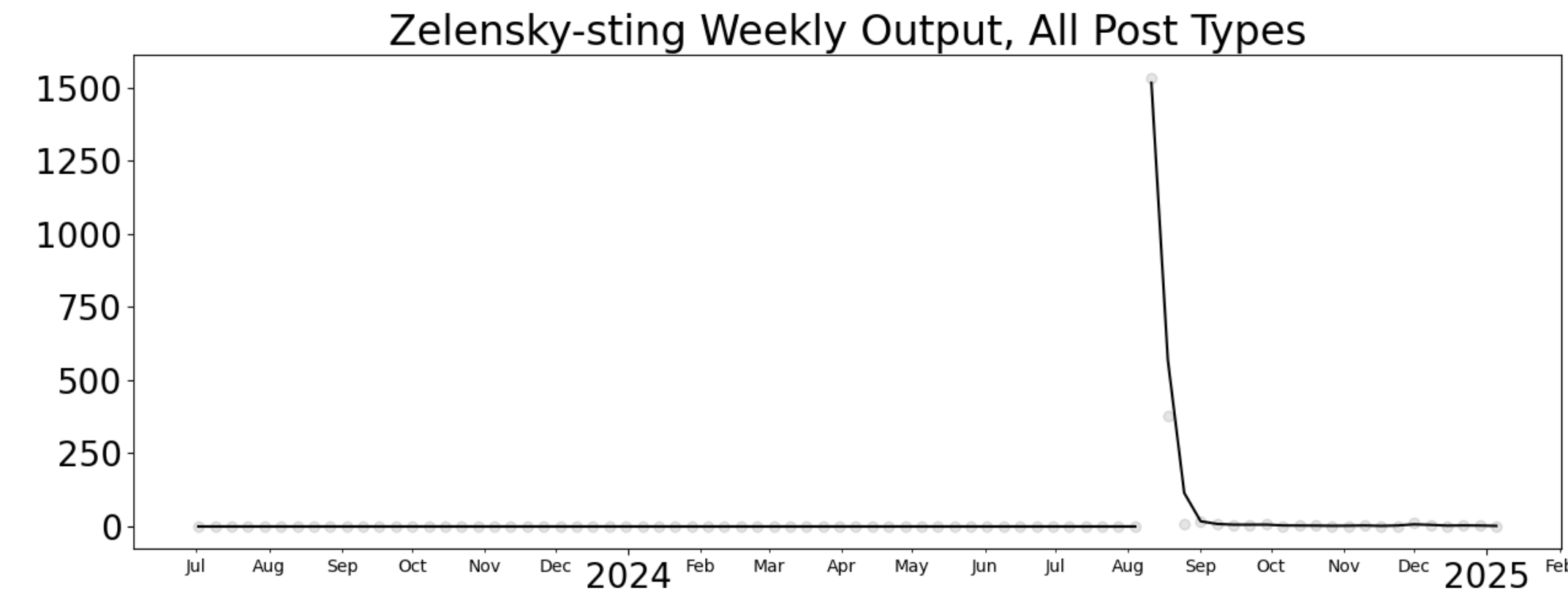

Zelensky-sting Weekly Output, All Post Types
1500
1250
1000
750
500
250
0
Jul
Aug
Sep
Oct
Nov
Dec
2024
Feb
Mar
Apr
May
Jun
Jul
Aug
Sep
Oct
Nov
Dec
2025
Feb

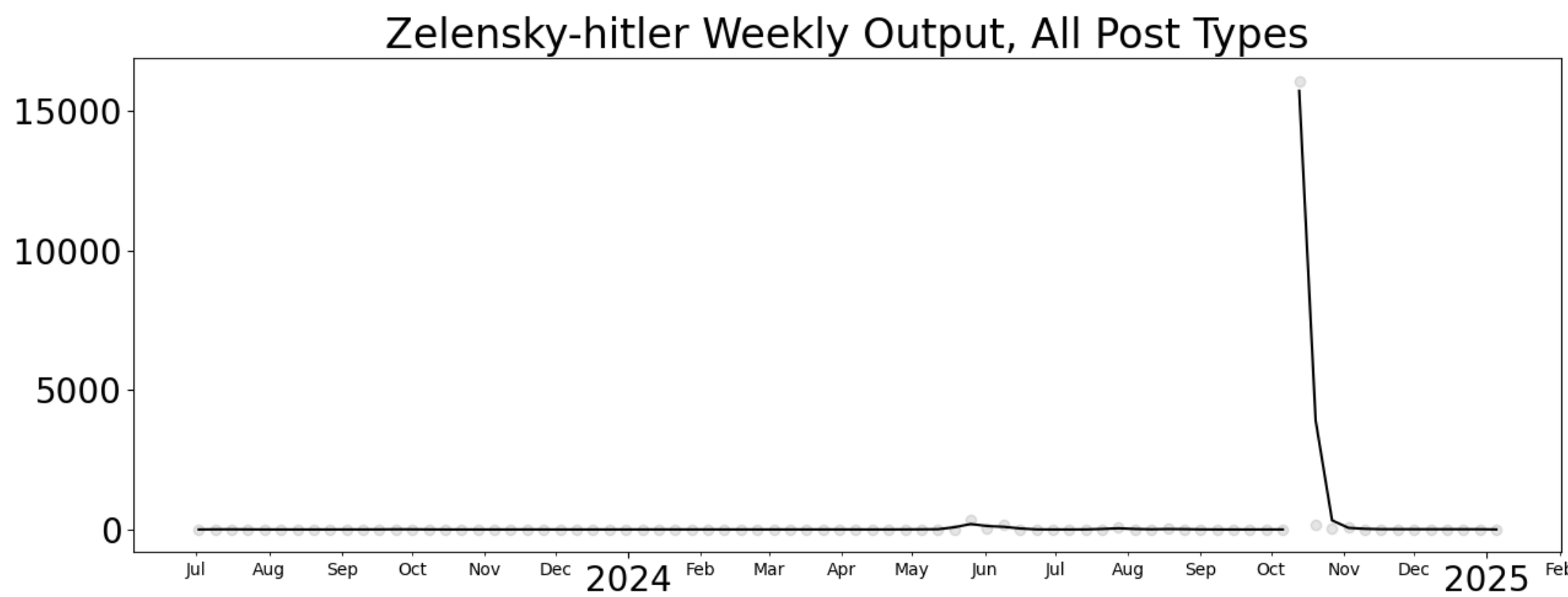

Zelensky-hitler Weekly Output, All Post Types
15000
10000
5000
0
Jul
Aug
Sep
Oct
Nov
Dec
2024
Feb
Mar
Apr
May
Jun
Jul
Aug
Sep
Oct
Nov
Dec
2025
Feb

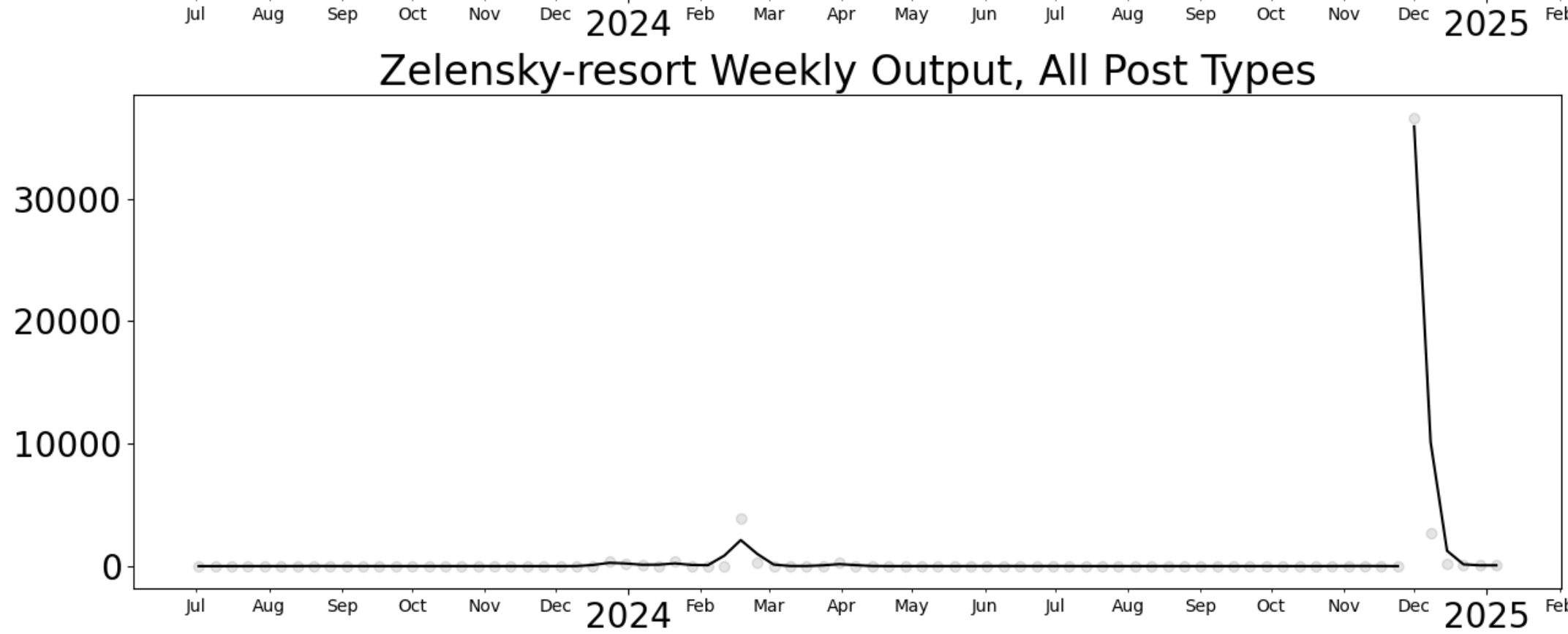

Zelensky-resort Weekly Output, All Post Types
30000
20000
10000
0
Jul
Aug
Sep
Oct
Nov
Dec
2024
Feb
Mar
Apr
May
Jun
Jul
Aug
Sep
Oct
Nov
Dec
2025
Feb

S3. LLM-Labeling Task

We randomly selected 40,000 posts from the full set of posts including Zelensky/Zelenskyy/Zelenska in our sampling period (July 1, 2023 to December 31, 2024).
We used a large language model as a text-classification tool. Each sampled post was assigned to one of three mutually exclusive categories: (1) posts that insinuate or explicitly state that Zelensky or Zelenska is financially corrupt or benefits from financial corruption, (2) posts that fact-check, debunk, or reject such allegations, and (3) posts unrelated to that metanarrative.

We used GPT-4o mini to classify the text of each sampled post using a fixed few-shot prompt. The model was shown only the text of the sampled post. It did not have access to attached images or video, linked content, user metadata, engagement statistics, or preceding and subsequent posts in a thread. It was given also given three examples of posts from the population with the correct label attached. Every post was evaluated on its own, with a single API call, in late February, 2025. The model was given no access to web-search and was restricted to giving responses of less than 150 tokens, with all other tuning parameters left at default.

The model was instructed to return only a single numeric label corresponding to the three categories above. The full prompt is reproduced below.

Prompt:

'''
CONTEXT
You will be provided with a social media post. The post can express one of the following ideas:
1) Insinuating or explicitly stating that Zelensky or Zelenska is financially corrupt or benefits from financial corruption (e.g., allegations about misuse of funds or luxury purchases).
2) Fact-checking or debunking or disagreeing with allegations of financial corruption against Zelensky (e.g., providing evidence or calling the claims false/fake or calling the claims 'propaganda').
3) Neither of the above (e.g., corruption-related discussion about people unrelated to Zelensky or Zelenska, general commentary about Zelensky being a bad leader, or unrelated topics).
INSTRUCTIONS
Classify the following post into one of these three categories. Your response must be a single number: 1, 2, or 3 corresponding to the three ideas described above. Avoid explanations or additional text.
EXAMPLES
Social Media Post: RT @Panchenko_X Today I'll show You the lifestyle of Ukrainian officials in France.
After the material was published in Ukrainian language, Zelenskyy started pressure on my colleagues from "Ukrainian Truth" Media (sponsored by Soros).
But we will fight!

And we need Your support.
Please like… https://t.co/h7LHNigNAk
Correct Response: 1
Social Media Post: RT @NinaByzantina How many zeroes is Zelensky's income declaration missing? pic.twitter.com/8zNnXHYeho
Correct Response: 1
Social Media Post: RT @Kime2Back ZELENSKY COMPRA EL CARRO D HITLER X 15 MILLONES D $ Y LO ESTACIONA JUSTO FUERA D SU OFICINA...Q PENSARÀN LAS VICTIMAS DEL HURACÀN HELENE CUANDO VEAN LO Q ZELENSKY ESTÀ HACIENDO CON LOS 8BILLONES QUE LE ENTREGÒ BIDEN MIENTRAS ELLOS MUEREN D MENGUA? x.com/goddeketal/sta…
Correct Response: 1
Social Media Post: RT @P_Kallioniemi There was no coup in 2014. The support for Nazis in Ukraine is miniscule. Zelenskyy hasn't bought villas. Ukraine hasn't sold weapons to Hamas. There was no genocide in Donbas. Zelenskyy banned churches and political parties connected to FSB. The election in Ukraine was postponed…
Correct Response: 2
Social Media Post: RT @RonFilipkowski A former FL police officer now living in Moscow is creating fake stories using AI while posing as local US newspapers. His fake story that Zelensky's wife bought a $4 million Bugatti was spread by millions of pro-Russia US social media accounts. bbc.com/news/articles/…
Correct Response: 2
Social Media Post: @omkolisnyk @Razvan1484598 @AndrewPerpetua Managing West's perception of Ukraine is far more important than you acknowledge & give him credit for. Sad but true.
An unfortunately large % of Westerners have bought into RF dezinformatsiya about biolabs, Nazis, cokehead Zelesky, billionaire Zelensky, irredeemably corrupt UKR
Correct Response: 2
Social Media Post: RT @DefiyantlyFree Zelensky coming here asking for our money and our ammunition while bashing Donald Trump in the media is called taxpayer subsidized election interference.
Correct Response: 3
Social Media Post: RT @govt_corrupt #WATCH: Just as support for the Ukraine war hits record lows, a children's hospital is targeted and Vladimir Zelensky and Justin Trudeau are blaming Russia without any evidence.
Remember when Ukraine bombed Poland 2 years ago? Remember the Reichstag Fire? https://t.co/YQpQuCjo8l
Correct Response: 3
Social Media Post: RT @Gerashchenko_en Instead of a thousand words pic.twitter.com/IFN3RBDYRv
Correct Response: 3
SOCIAL MEDIA POST FOR YOU TO CLASSIFY
Social Media Post: {message}
'''

Figure S1 presents a confusion matrix for the LLM coded responses to a randomly selected sub-sample of 299 posts. For these posts, we had two research assistants independently code the full set of 299 posts using the same instructions as were provided

to the LLM. They then compared and aligned their responses to disagreements. These aligned answers represent the human response presented in the matrix.

Relative to the LLM, the human encoders had access to much more information. They had access not only to the message, but also access to link to the post on X, which they were instructed to follow. Thus, they were able to access any media shared in the post, any previews for links included in the post, and substantial information about the conversational context in which the post appeared, such as (for example) the content of posts to which the focal post was replying. As such, we interpret the human-coded response as approximating ground-truth for the actual underlying character of the post, rather than a best guess available from seeing only the text.

There was very little confusion between responses 1 (corruption) and 2 (fact checking). Of the 75 posts that the LLM encoded as implicating corruption, 65 (87%) were coded the same way by the human coders, while only 1 (1.3%) was coded as fact-checking. While of the of the 32 posts that the LLM coded as involving fact-checking, the humans coded one to actually implicate corruption (3%), with the rest (97%) coded as fact-checking. The LLM was substantial weaker at judging whether the post actually revolved around specific allegations of corruption, at all. Of the 192 posts that the LLM coded as unrelated to the specific topic of Zelensky's corruption, the humans coded 46 (24%) to actually be (often implicit) references to his corruption. Most of these misses come from the LLM not having access to the context around the posts, leaving it without sufficient information to infer correctly the underlying implication.

If the imperfections in LLM classification simply introduce noise in metric of the corruption metanarrative, it will make our estimates of the impact less precisely estimated. If, however, the LLM is particularly poor/good at recognizing the specific sort of narratives that Storm-1516 pushes, it would result in an under/over-estimation of the impact of the Storm-1516 campaign.

Figure S1. Confusion Matrix

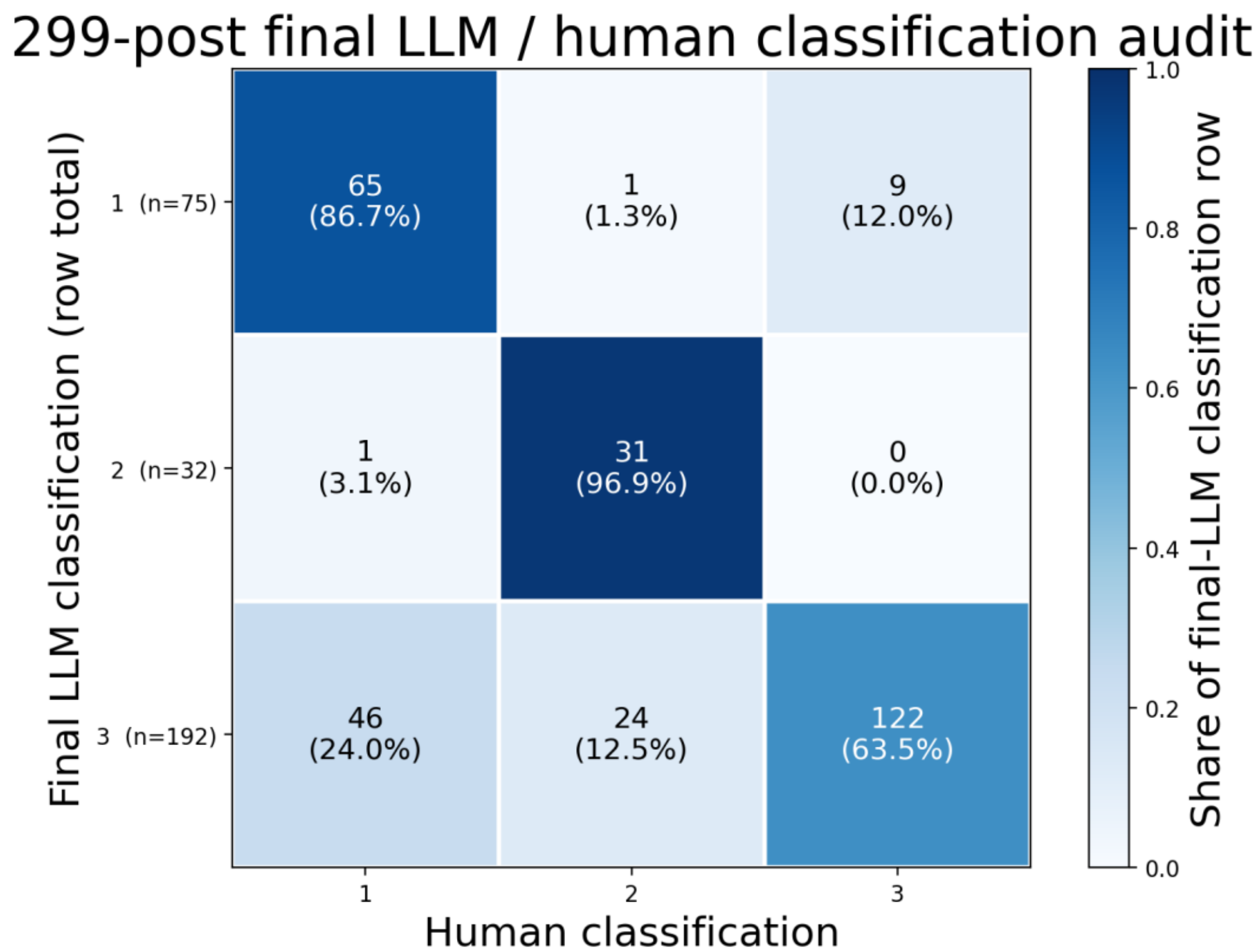

S4. High-Follower Sub-Sample

This distribution of follower counts for messages in the overall Zelensky conversation have the following deciles

Table S1. Sender Followers Count Distribution across Zelensky-Related Messages

**Decile**	**Sender Follower Count**
0.0	0
0.1	0
0.2	70
0.3	183
0.4	335
0.5	563
0.6	899
0.7	1,441
0.8	2,421
0.9	4,743
1.0	22,569,971

Removing all messages from accounts with follower counts in the bottom two deciles (<71 followers), we replicate the two analyses apply to X, the direct keyword narrative share and the corruption metanarrative share. Those results are presented in Table S2.

Table S2. Event Study Analysis of the Relationship between Narrative Introduction and Proxies of Narrative Impact, Removing Low-Follower Accounts

Time Period	X Narrative Share	X Corruption Share
Base Rate	0.000	0.115**
	(0.001)	(0.008)
0 weeks after	0.089**	0.090**
	(0.020)	(0.011)
1 week after	0.008**	-0.004
	(0.003)	(0.015)
2 weeks after	0.004	-0.008
	(0.003)	(0.013)
3 weeks after	0.004	0.015
	(0.004)	(0.019)
4 weeks after	0.001	0.015
	(0.004)	(0.024)

Note: * $p < 0.05$, ** $p < 0.01$

S5. Window Length Robustness

We replicate the analysis for all three dependent variables, while varying the length of the pre-treatment window from 2 to 6 weeks, and present the results in Table S3. The main specification from the body (4w) is included to ease comparisons.

Table S3. Robustness to pre-treatment window length.

Time Period	Outcome	Pre-Treat 2w	Pre-Treat 3w	Pre-Treat 4w	Pre-Treat 5w	Pre-Treat 6w
0 weeks after	Google Trend Share	0.0531*	0.0529*	0.0532*	0.0531*	0.0532*
		(0.0240)	(0.0240)	(0.0240)	(0.0241)	(0.0241)
	X Corruption Share	0.0727**	0.0738**	0.0677**	0.0649**	0.0677**
		(0.0142)	(0.0119)	(0.0117)	(0.0116)	(0.0119)
	X Narrative Share	0.0762**	0.0762**	0.0760**	0.0759**	0.0760**
		(0.0168)	(0.0168)	(0.0168)	(0.0168)	(0.0169)
1 week after	Google Trend Share	0.0247	0.0245	0.0248	0.0247*	0.0248*
		(0.0131)	(0.0127)	(0.0125)	(0.0123)	(0.0123)
	X Corruption Share	-0.0011	-0.0001	-0.0061	-0.0090	-0.0062
		(0.0165)	(0.0143)	(0.0132)	(0.0125)	(0.0127)
	X Narrative Share	0.0089*	0.0089**	0.0087**	0.0087**	0.0087**
		(0.0038)	(0.0032)	(0.0028)	(0.0027)	(0.0026)
2 weeks after	Google Trend Share	0.0076	0.0074	0.0077*	0.0076**	0.0077**
		(0.0050)	(0.0038)	(0.0032)	(0.0027)	(0.0024)
	X Corruption Share	-0.0008	0.0002	-0.0058	-0.0086	-0.0059
		(0.0149)	(0.0123)	(0.0123)	(0.0118)	(0.0112)
	X Narrative Share	0.0158	0.0158	0.0156	0.0156	0.0156
		(0.0097)	(0.0095)	(0.0094)	(0.0094)	(0.0094)
3 weeks after	Google Trend Share	0.0038	0.0037	0.0039	0.0039*	0.0040*
		(0.0044)	(0.0031)	(0.0024)	(0.0020)	(0.0017)
	X Corruption Share	0.0247	0.0257	0.0197	0.0169	0.0196
		(0.0188)	(0.0173)	(0.0173)	(0.0175)	(0.0175)
	X Narrative Share	0.0036	0.0036	0.0034	0.0033	0.0034
		(0.0048)	(0.0041)	(0.0037)	(0.0034)	(0.0032)
4 weeks after	Google Trend Share	0.0027	0.0026	0.0029	0.0028	0.0029
		(0.0054)	(0.0042)	(0.0034)	(0.0029)	(0.0025)
	X Corruption Share	0.0121	0.0132	0.0071	0.0043	0.0071
		(0.0203)	(0.0189)	(0.0190)	(0.0192)	(0.0187)
	X Narrative Share	0.0033	0.0034	0.0031	0.0031	0.0032
		(0.0048)	(0.0041)	(0.0037)	(0.0034)	(0.0032)
Base Rate	Google Trend Share	0.0009	0.0010	0.0007	0.0008	0.0007
		(0.0036)	(0.0026)	(0.0019)	(0.0016)	(0.0013)
	X Corruption Share	0.0972**	0.0962**	0.1022**	0.1050**	0.1023**
		(0.0107)	(0.0073)	(0.0074)	(0.0066)	(0.0061)
	X Narrative Share	0.0003	0.0003	0.0005	0.0006	0.0005
		(0.0025)	(0.0018)	(0.0014)	(0.0011)	(0.0009)

*Note: * p < 0.05, ** p < 0.01*

S6. Narrative-by-Narrative Contrasts

The results in the body pool together the contrasts between the pre-treatment period and the weeks of or after treatment for all eleven narratives. Table S4 presents the narrative-by-narrative contrasts for the week of (Week 0) and the week after treatment (Week 1). The final row in bold presents the average across all eleven narratives. These average results are presented for comparison, and they replicate the main results from the body.

Table S4. Simple Contrasts with Base Period by Narrative and Overall

Narrative	X Narrative Share Week 0 Contrast	X Narrative Share Week 1 Contrast	X Corruption Share Week 0 Contrast	X Corruption Share Week 1 Contrast	Google Trend Share Week 0 Contrast	Google Trend Share Week 1 Contrast
Bugatti	0.202	0.008	0.116	-0.035	0.300	0.021
Cartier	0.102	0.014	0.076	-0.029	0.043	0.020
Casino	0.084	0.003	0.077	0.009	0.031	0.027
Dubai	0.004	0.000	-0.004	0.033	0.000	0.000
Highgrove	0.033	0.002	0.053	-0.017	0.097	0.009
Hitler	0.035	0.000	0.088	-0.045	0.008	0.000
Resort	0.060	0.001	0.069	0.021	0.027	0.006
Sting	0.006	0.001	0.047	-0.069	0.043	0.008
Villa	0.131	0.014	0.051	0.049	0.002	0.028
Villa_2	0.125	0.022	0.076	-0.026	0.033	0.006
Yacht	0.054	0.031	0.096	0.040	0.000	0.148
**Average**	**0.076**	**0.009**	**0.068**	**-0.006**	**0.053**	**0.025**

S7. Alternative Regression Forms with Limited Dependent Variables

The main specification presented in the body of the paper models the relationship between treatment timing and narratives shares as an ordinary least squared regression. Although OLS is t best linear model of the conditional expectation, it has some unattractive properties in setting where the dependent variable is, in fact, bounded. To test the robustness of our results to alternative models that explicitly model this boundedness, we estimated the parallel versions of our model under the assumption of the Beta regression and the continuous Logit regression.

Unfortunately, both these models suffer from a parallel problem to OLS, where our data, in practice, do not quite fit the formal requirements of the model, as neither can handle exact zeros (or ones) in the dependent variable. For that reason, we first transform the dependent variable using the Smithson-Verkuilen (Psych Meth, 2006) adjustment to move the data into the open unit interval. Otherwise, we run the exact same specification as in the body.

Table S5 displays the results of these analyses, for the key coefficients (0-week and 1-week), as well as baseline rates and predicted change in the shares for those models.

Table S5. Results with Limited Dependent Variable Models

Outcome	Model	Period	Coef.	Std. Error	Baseline Share	Predicted Share	Discrete Change
X Narrative Share	Logit FE	0 weeks after	2.428	0.289	0.001	0.056	0.055
X Narrative Share	Logit FE	1 week after	0.723	0.156	0.001	0.007	0.006
X Narrative Share	Beta FE	0 weeks after	2.358	0.154	0.003	0.072	0.069
X Narrative Share	Beta FE	1 week after	0.700	0.206	0.003	0.011	0.008
X Corruption Share	Logit FE	0 weeks after	0.630	0.104	0.095	0.168	0.073
X Corruption Share	Logit FE	1 week after	-0.060	0.129	0.095	0.090	-0.005
X Corruption Share	Beta FE	0 weeks after	0.596	0.114	0.102	0.173	0.071
X Corruption Share	Beta FE	1 week after	-0.039	0.134	0.102	0.098	-0.004
Google Trend Share	Logit FE	0 weeks after	1.721	0.385	0.001	0.027	0.026
Google Trend Share	Logit FE	1 week after	1.235	0.259	0.001	0.015	0.014
Google Trend Share	Beta FE	0 weeks after	1.589	0.211	0.004	0.040	0.035
Google Trend Share	Beta FE	1 week after	0.975	0.221	0.004	0.020	0.015

S8. Permutation Estimates of Hypothesis Tests

In the body, we use standard heteroskedasticity-robust asymptotic standard errors. But as the sample size is quite small (99 observations). We also conducted a 500-fold permutation exercise where it randomly allocated the labels for treatment/control weeks within each narrative. We then calculated the fraction of these permutations where the estimated coefficient would be at least as large as our true estimates. That fraction is recoded as the permutation p-value.

Table S6 presents the results of that exercise for our 6 key parameters. In all three cases, the week-0 parameters remain strongly statistically significant. But the week-1 results are quite fragile, with only the Google Trend results showing (marginal) significance.

Table S6. p-value Estimates for Key Coefficients -- Asymptotic and Permutation Approaches

Outcome	Period	Observed Coefficient	Robust SE	Asymptotic p-value	Permutation p-value
X Narrative Share	0 weeks after	0.076	0.017	<0.001	0.002
X Narrative Share	1 week after	0.009	0.003	0.003	0.427
X Corruption Share	0 weeks after	0.068	0.012	<0.001	0.002
X Corruption Share	1 week after	-0.006	0.013	0.643	0.715
Google Trend Share	0 weeks after	0.053	0.024	0.03	0.002
Google Trend Share	1 week after	0.025	0.012	0.05	0.068

S9. GUIDE-LLM: A reporting checklist for large language models in behavioural science

The GUIDE-LLM checklist provides a standardized framework for reporting studies that use large language models (LLMs) in the behavioral and social sciences. It aims to promote transparency, reproducibility, and ethical accountability across all stages of LLM-based research.

How to complete the checklist:
Fill in each item with brief, specific information about how LLMs were used in your study. Where an item does not apply, write "N/A" and, if helpful, note why. If multiple LLMs were used for different purposes, complete the relevant sections separately for each model. You may refer to sections or appendices in your manuscript rather than repeating text.

**Scope of LLM use**	**Answer**
**Item A.1:** LLMs were used in this project for:<br>**Explanation:** Briefly describe how and for what purposes LLMs were used in the study. This may include one or multiple stages of the research workflow, depending on the project's design and aims. The following examples illustrate common use cases:<br>● **Research design** (e.g., hypothesis generation, literature search, or creating surveys/stimuli).<br>● **Data processing** (e.g., transcription, translation, data extraction, or data cleaning).<br>● **Analysis** (e.g., data labeling, summarization, pattern detection, statistical analysis, or code generation).<br>● **LLM as research object** (e.g., studying LLM behavior, benchmarking LLMs, or bias assessment of LLMs).<br>● **LLM as an evaluator** (e.g., evaluating model output, assessing the quality of human labeling/annotation)<br>● **Participant-facing settings** (e.g., LLM used as an intervention, studying human interactions with LLM chatbots).<br>● **Communication** (e.g., paper writing, editing, or reviewing).<br><br>Depending on the specific use case described here, different checklist items may later be relevant, and, in many cases, it may be necessary that later items in the checklist are reported separately for each use case.	1. Analysis 1: GPT-4o mini was also used to perform the classification exercise, which is discussed in the methods section and, in detail, in section S3 of the SI to perform the classification exercise, which is discussed in the methods section and, in detail, in section S3 of the SI.<br>2. Analysis 2: Codex, with GPT 5.4, was used as a coding assistant for the implementation of simple visualizations and standard statistical procedures.<br>3. Communication: GPT-5.4 and Grammarly were used for limited formatting and grammar review.

**Item A.2:** Degree of automation (human-in-the-loop vs. fully automated):<br><br>**Explanation:** Indicate how much human oversight was involved. Specify whether each output was reviewed, edited, or approved by a person, or whether outputs were used automatically without supervision. For participant-facing tasks, state whether humans checked outputs before showing them to participants or whether participants interacted with the LLM directly. Specify who provided oversight (e.g., student assistant, expert, PI).	1. Automated, but evaluated against human coders<br>2. Fully human-in-the-loop, with interactive instruction and final code review<br>3. Fully human-in-the-loop with final word choices fully controlled by human.

Model/system details	Answer
**Item B.1:** Model name, including provider, model size, exact version/ID, date of access, and source link (if possible):<br><br>**Explanation:** Report the exact model names (including provider, version, and date accessed). Avoid generic labels like "ChatGPT" or "GPT-4"; instead, use detailed model names such as *"GPT-4o-mini-2024-12-17 (OpenAI)"* or *"Llama-3.1-8B (Meta; accessed via HuggingFace in May 2025)"*. For locally deployable models, please also enter a source link (e.g., the URL to the HuggingFace page). If multiple models were tested, it is encouraged to name them and briefly explain which one was used in the final study and why. When multiple models served different purposes, specify their respective roles, consistent with your response to Item A.1.	1. GPT-4o mini<br>2. Codex with GPT 5.4<br>3. GPT 5.4 and Grammarly
**Item B.2:** Model access (e.g., API, web interface, local) and context mode (e.g., chat mode or separate calls):<br><br>**Explanation:** Note how you accessed the models (e.g., API, web interface, local installation) and whether you used LLMs in **chat mode** (ongoing conversation) or **stateless** mode (separate prompts). Mention the exact API name and version, since different access modes may influence responses (e.g., due to differences in model routing).	1. API<br>2. Codex for Linux<br>3. Chat mode
**Item B.3:** Relevant LLM configurations reported (as applicable), such as temperature, max tokens, seed, and number of runs:<br><br>**Explanation:** List any configuration settings that may affect outputs, such as:<br>• **temperature** (which controls randomness of the model's output)<br>• **Sampling parameters** such as **top_k, top_p, max tokens** (which limit the candidate token set or enforce length constraints)<br>• P**enalties** that discourage repetition (e.g., a frequency penalty to reduce the likelihood of tokens proportional to how often they have already appeared; a presence penalty reduces the likelihood of any token that has appeared at least once)	1. We used GPT-4o mini to classify the text of each sampled post using a fixed few-shot prompt. The model was shown only the text of the sampled post. It did not have access to attached images or video, linked content, user metadata, engagement statistics, or preceding and subsequent posts in a thread. It was given

<table>
<tr>
<td>

- **Stop sequences** (which halt generation when such a specified sequence is produced; e.g., ["\n\n", "END"]).
- **Number of completions or runs** (which is often used to capture variability in outputs across repeated generations)
- **Quantization level** (e.g., FP16, INT8, INT4) to change numerical precision beyond the default
- **Reasoning-related settings**, such as whether a specific structured reasoning was enabled, the specified reasoning effort level (e.g., low/medium/high or numerical settings that influence the depth of the reasoning), and any compute or inference budget constraints tied to the chosen reasoning mode

</td>
<td>

also given three examples of posts from the population with the correct label attached. Every post was evaluated on its own, with a single API call, in late February, 2025. The model was given no access to web-search and was restricted to giving responses of less than 150 tokens, with all other tuning parameters left at default.

2. NA
3. NA

</td>
</tr>
<tr>
<td>

Item B.4: Customization:

Explanation: Check and describe any modifications or extended capabilities incorporated into your LLM setup beyond standard inference. This includes, but is not limited to:

- **Fine-tuning** (e.g., via LoRA; Low-Rank Adaptation) used to adapt a pretrained model to domain-specific data.
- **Retrieval-augmentation generation (RAG), where** the model retrieves relevant information from external sources (e.g., databases or document collections) during inference.
- **Automated prompt optimization** (e.g., DSPy) that treat prompts as trainable parameters.
- **Web search integration**, indicating whether the LLM was able to access and retrieve information from the Internet.
- **Agentic workflows**, including multi-step reasoning processes or delegated actions such as tool/function calling (e.g., via LangChain, AutoGPT, CrewAI).
- **Post-training refinements**, including alignment or optimization techniques used to adjust model behavior after pretraining (e.g., reinforcement learning from human feedback (RLHF), direct preference optimization (DPO)).

  The goal is to specify any added customizations or provider-specific features that meaningfully shape system behavior in order to enable others to understand and accurately reproduce your setup.

</td>
<td>

☐ Base model
☐ Fine-tuning
☐ RAG (retrieval-augmented)
☐ Automated prompt optimization
☐ Tool/function calling
☐ Web search
☐ Agentic workflows
☐ Other adaptations (e.g., safety mechanisms)

Description:

</td>
</tr>
<tr>
<td>

Item B.5: Did the LLM session(s) include persistent memory across interactions?

Explanation: Indicate whether the LLM could “remember” previous conversations (i.e., had persistent memory). Unless such memory is disabled, there may also be spillover effects from other chat windows

</td>
<td>

☐ Yes
☐ No
☐ N/A

</td>
</tr>
</table>

or prior conversations, which can influence outputs even when not intended.	

Prompts	Answer
**Item C.1:** Exact prompt(s) reported:<br><br>**Explanation:** Whenever possible, include the **exact** text of prompts you used, including in-context examples or demonstrations provided to the LLM. Even small wording changes, formatting, or ordering of examples can substantially affect outputs. If full prompts cannot be shared (e.g., due to privacy or length), include a redacted or representative example or link to the full prompt in a repository (e.g., OSF, GitHub). If prompts were iteratively refined during the study, document the nature and extent of these refinements, including whether changes were pre-specified or made in response to interim outputs, to ensure transparency regarding potential optimization or selective reporting.	1. Full prompt included in SI3<br>2. NA<br>3. NA
**Item C.2:** System-wide instructions (if any):<br><br>**Explanation:** Note any *system-level* instructions that guide the model's general behavior (e.g., "You are a helpful assistant."). These are commonly not directly visible but can be accessed through the API.	NA

Data inputs & privacy	Answer
**Item D.1:** Handling of personal or sensitive data (if any) (e.g., consent for data processing):<br><br>**Explanation:** If any personal, sensitive, or identifiable data were processed, describe how they were handled in compliance with ethical standards and data protection laws. Researchers should indicate whether participants explicitly consented to their data being analyzed with an LLM, particularly when proprietary, cloud-based models are used. Such processing typically involves transferring data to a private company that may retain them indefinitely, which raises additional ethical and legal considerations. Beyond consent, describe how sensitive or identifiable data were handled (e.g., de-identification, anonymization, masking) and whether the LLM provider offers safeguards such as excluding inputs from training or storage. Clarify where data were stored or processed and how applicable legal/ethical requirements were met. If relevant, address cross-border transfers, as data may be stored in jurisdictions with different privacy laws (e.g., EU vs. US), with implications for compliance with GDPR, HIPAA, or other frameworks.<br><br>For context, some providers (e.g., OpenAI) may log or inspect prompts even when the data are not used for model training. For sensitive datasets, zero-retention configurations may be required (e.g., the MIMIC datasets can only be used with OpenAI models if a zero-retention checkpoint is enabled).	NA

**Validation & interpretation**	**Answer**
**Item E.1:** Human validation of LLM outputs:<br><br>**Explanation:** If relevant, describe whether and how human reviewers examined the model's outputs, and the degree of independence they had in doing so. Specify the reviewers' roles (e.g., domain experts, research assistants, subject-matter specialists) and relevant expertise, as well as how many reviewers participated and how their work was organized. Indicate whether outputs were independently annotated, double-checked by multiple reviewers, or merely approved or edited post-hoc by a lead author or investigator.<br><br>Clarify what dimensions of performance were examined. These may include known performance metrics from ML/AI such as accuracy or other metrics like citation correctness, hallucination detection, agreement or inter-rater reliability. State whether qualitative judgments, quantitative metrics, or both were used. If outcome assessment required subjective interpretation, describe assessor qualifications, instructions provided, and relevant demographics.<br><br>Describe the selection procedure for the reviewed outputs—whether all outputs were examined, a random sample was drawn, or specific cases (e.g., rare events or high-stakes responses) were oversampled to capture potential rare or critical errors. Further report how reviewers were trained or instructed, what criteria or rating scales they used, and how disagreements were resolved. For multi-reviewer settings, provide any inter-rater or inter-assessor reliability statistics (e.g., Cohen's κ or Krippendorff's α). Finally, note whether reviewer feedback was used purely for validation or also to refine prompts, retrain models, or adjust study procedures.	X Yes<br>☐ No<br>☐ N/A<br><br>Description:<br><br>1. Random subset of labelled posts were compared to human labels.
**Item E.2:** Describe any relevant post-processing (e.g., filtering in case of format mismatches, unit conversions, etc.):<br><br>**Explanation:** Describe any steps you took to clean or reformat LLM outputs (e.g., converting "positive/neutral/negative" to numeric codes, handling missing values, removing malformed entries). State how you handled inconsistent or unusable outputs and whether corrections were made with an automated script or manually. For example, when generating quantitative estimates (e.g., word counts, probabilities, or durations), the model may return values embedded in free text (e.g., "3.5 seconds") that require parsing and conversion into standardized numerical units. Post-processing steps should be described clearly, including how formatting errors, null responses, or inconsistent output structures were handled, whether automated scripts or manual corrections were used, and whether any data were excluded or reinterpreted as a result. Report how outputs were selected, extracted, or consolidated when multiple generations were produced.	NA

**Reproducibility**	**Answer**

**Item F.1:** Code/notebooks/scripts for LLM calls shared:<br><br>**Explanation:** Indicate whether you have shared materials such as code, prompts, logs, or transcripts. Make sure sensitive information (e.g., API keys, private data) is removed. For code, make sure to add a README file.	☐ Yes<br>☐ No<br>☐ N/A<br><br>Link/DOI:

**Competing interests**	**Answer**
**Item G.1:** Funding, support, or other relevant relationships (including in-kind access to compute or models, or professional affiliations):<br><br>**Explanation:** Disclose any current or past funding, support, or other relevant relationships with entities that have a financial interest in LLMs (this includes not just AI companies like OpenAI, Anthropic, but also tech companies developing or investing in AI, e.g., Google, Meta, Microsoft). This could include (but is not limited to): research funding from or collaborative research with a company with an interest in LLMs for this project or any other project within the past years; in-kind access to compute or models; current or former professional affiliations with a company with an interest in LLMs; personal investments (e.g., stocks) in companies with an interest in LLMs; familial relationship with an employee of a company with an interest in LLMs; etc. Disclose these relationships regardless of whether or not you believe they impacted the research.	☐ Yes. Description:<br>X No

**Optional items**	**Answer**
Justification for the specific LLM choice along the following dimensions:<br><br>**Explanation:** Briefly explain why you chose this specific model for your study and thereby explain why the selected model is suited to the research needs. Consider:<br>● **Performance:** Explain why this model's accuracy or size was appropriate for the research task, and, if relevant, consider compute cost, scalability, and real-time feasibility.<br>● **Transparency:** Was the model open-weight or closed-weight? Do we know what training data the model was exposed to or what the training objective was? If not, what are the implications for interpreting results? In terms of openness, different levels can be distinguished, in which only the model weights are publicly available ("open-weight") versus LLMs where the entire training process is available to allow for further customization and transparency of the LLM itself ("open-source").<br>● **Reproducibility:** Can the model be shared (so results can be reproduced in the future), or is access limited to proprietary/cloud	X Performance – Description:<br><br>Performance needed to be sufficiently good, both in terms of precision/recall as well as in terms of speed.<br><br>☐ Transparency – Description:<br><br>☐ Reproducibility – Description:<br><br>☐ Ethical considerations – Description:

services (so the access to the model may be altered, restricted, or discontinued)?<br>• **Ethical considerations:** Are there ethical reasons (including safety) for the model choice, such as running a model locally to avoid sharing sensitive data?<br>• **Other determinants** can be the cost or ease-of-use (e.g., due to accessibility of certain models via university infrastructures), if their benefits outweigh trade-offs in other dimensions.	X Others (e.g., cost, ease-of-use): – Description:<br><br>Cost was important, leading us to use the mini model, since performance was sufficiently good.
Discussion of the rationale for the prompt design:<br>**Explanation:** Explain how you designed your prompts. For example, indicate whether you used a structured format (e.g., explicit task description, definitions, step-by-step instructions, and output constraints), followed established prompt engineering guidelines or prior literature, adapted prompts from earlier studies, or relied on automated prompt optimization tools. Clarify whether the design was iterative (e.g., refined through pilot testing or error analysis), whether few-shot examples were included and how they were selected, and whether prompts were standardized across models to ensure comparability.	X Yes. Description:<br><br>Exploratory prompting and evaluation on small samples of the data was done before settling on the final prompt.<br><br>☐ No<br>☐ N/A
Comparison against other methods/LLMs:<br>**Explanation:** If you compared different models, prompts, or methods, describe how you made the comparison and what criteria you used (e.g., accuracy). State whether you used the same inputs across methods and how you ensured fair comparison. If you considered but excluded alternative methods/LLMs, briefly explain why. If such comparisons are made, report the exact benchmarking procedure and the level of agreement between methods. If other LLMs or methods were considered but not used, briefly explain the rationale (e.g., cost, access, performance, or ethical considerations).	☐ Yes. Description:<br>X No<br>☐ N/A
Training data leakage risks addressed:<br>**Explanation:** Note whether you considered the chance that your evaluation materials (e.g., test data, questionnaires) were already part of a model's training data. If relevant, describe steps taken to mitigate or detect leakage, such as novelty checks, re-phrasing or re-structuring items, or using hold-out materials not widely available.	☐ Yes<br>☐ No<br>X N/A<br><br>Novel question
Potential risk of bias or other systematic differences in LLM behavior that could affect the study's conclusions?<br>**Explanation:** Reflect on whether the LLM might perform differently across groups, languages, or contexts in ways that could affect your conclusions. If you checked for or mitigated such bias, describe how (e.g., subgroup analyses, balanced sampling, sensitivity checks). Where applicable, researchers may consult dedicated bias and fairness checklists for structured guidance on identifying and addressing these issues.	☐ Yes. Description:<br>X No

Conversation transcripts:<br><br>**Explanation:** For studies where participants or researchers interacted directly with an LLM (e.g., chat-based interventions, behavioral experiments, or coding tasks), provide anonymized transcripts or representative examples of the interactions. Redact any personally identifiable or sensitive information.	☐ Yes, shared without sensitive information.<br>Location:<br>☐ No. Reason for not sharing:<br>X N/A
Discuss relevant ethical implications of the research:<br><br>**Explanation:** Briefly discuss the broader ethical considerations of your study beyond procedural compliance (e.g., IRB approval). Address potential risks and safeguards, including:<br>● **Participant well-being:** foreseeable harms (emotional, reputational, financial, environmental), inclusion/exclusion criteria, effects on vulnerable groups, accessibility, use of deception and debriefing, autonomy and informed consent (including clear disclosure of AI involvement).<br>● **Fairness and equity:** whether outputs or outcomes may reflect or amplify bias or discrimination; note any subgroup analyses or mitigation strategies.<br>● **Safety:** broader risks (such as from agentic or autonomous behavior), or safeguards for harmful or adverse outputs (e.g., moderation layers, content filters, human-in-the-loop review, escalation protocols, red-teaming, dual use).	We used public expressive data with no behavioral interventions. Minimal risk.
Computational resources (e.g., API call counts, tokens, financial costs, or compute time):<br><br>**Explanation:** Report the computational resources required for the study. This may include API call counts, total tokens processed, financial costs, runtime, number and type of GPUs/CPUs used, and any other relevant compute metrics.	1. 40,000 API calls

How to cite:
Feuerriegel, S Barrie, C., Crockett, M. J., Globig, L. K., McLoughlin, K. L., Mirea, D.-M., Spirling, A., Yang, D., ..., Rathje, S., & Ribeiro, M. H. (2026). *A reporting checklist for large language models in behavioural science*. In: *Nature Human Behaviour*.. DOI: 10.1038/s41562-026-02492-7

SI References

Smithson, M., & Verkuilen, J. (2006). A better lemon squeezer? Maximum-likelihood regression with beta-distributed dependent variables. *Psychological Methods, 11*(1), 54–71. https://doi.org/10.1037/1082-989X.11.1.54
